# The chromatin remodeling protein ATRX positively regulates IRF3-dependent type I interferon production and interferon-induced gene expression

**DOI:** 10.1371/journal.ppat.1010748

**Published:** 2022-08-08

**Authors:** Anne-Charlotte Stilp, Myriam Scherer, Patrick König, Axel Fürstberger, Hans A. Kestler, Thomas Stamminger

**Affiliations:** 1 Institute of Virology, Ulm University Medical Center, Ulm, Germany; 2 Institute of Medical Systems Biology, Ulm University, Ulm, Germany; University of Wisconsin-Madison, UNITED STATES

## Abstract

The chromatin remodeling protein alpha thalassemia/mental retardation syndrome X-linked (ATRX) is a component of promyelocytic leukemia nuclear bodies (PML-NBs) and thereby mediates intrinsic immunity against several viruses including human cytomegalovirus (HCMV). As a consequence, viruses have evolved different mechanisms to antagonize ATRX, such as displacement from PML-NBs or degradation. Here, we show that depletion of ATRX results in an overall impaired antiviral state by decreasing transcription and subsequent secretion of type I IFNs, which is followed by reduced expression of interferon-stimulated genes (ISGs). ATRX interacts with the transcription factor interferon regulatory factor 3 (IRF3) and associates with the IFN-β promoter to facilitate transcription. Furthermore, whole transcriptome sequencing revealed that ATRX is required for efficient IFN-induced expression of a distinct set of ISGs. Mechanistically, we found that ATRX positively modulates chromatin accessibility specifically upon IFN signaling, thereby affecting promoter regions with recognition motifs for AP-1 family transcription factors. In summary, our study uncovers a novel co-activating function of the chromatin remodeling factor ATRX in innate immunity that regulates chromatin accessibility and subsequent transcription of interferons and ISGs. Consequently, ATRX antagonization by viral proteins and *ATRX* mutations in tumors represent important strategies to broadly compromise both intrinsic and innate immune responses.

## Author summary

ATRX is a member of a family of chromatin remodeling proteins required for deposition of the histone variant H3.3 at specific genomic regions. This is important to maintain silencing at these sites. Furthermore, ATRX represents a component of PML nuclear bodies (PML-NBs) which are considered as enigmatic nuclear protein accumulations exhibiting a tight link to cell-intrinsic restriction of viral infections. Previous studies demonstrated that many viruses target ATRX by either displacement or degradation. So far, it is believed that this serves to alleviate ATRX-instituted silencing of viral gene expression. Our results reveal a novel and unexpectedly broad function of ATRX as a co-activator of the innate immune response. We show that ATRX is required for both DNA and RNA sensing pathways to activate interferon (IFN) gene expression as well as for upregulation of a distinct set of interferon-stimulated genes. Assessment of chromatin accessibility detected that IFN acts as a switch to regulate the function of ATRX in heterochromatin remodeling. ATRX positively modulates chromatin accessibility specifically upon IFN signaling, thereby affecting promoter regions with recognition motifs for AP-1 family transcription factors. Loss of ATRX due to viral infection or due to tumor mutations may thus broadly compromise cellular innate immunity.

## Introduction

The production of type I interferons (IFNs) is a principal part of the innate immune system that enables recognition and counteraction of pathogens immediately upon entry into the host cell. Viral nucleic acids thereby serve as so-called pathogen-associated molecular patterns (PAMPs) that are sensed by diverse pattern recognition receptors (PRRs) including DNA sensors, such as cyclic GMP-AMP (cGAMP) synthase (cGAS), as well as RNA sensors, such as retinoic acid-inducible gene-I (RIG-I) [1–3]. Once activated, these PRRs induce signaling cascades that eventually lead to the production of type I IFNs. Upon binding of cGAS to DNA, cGAS catalyzes the production of the non-canonical cyclic dinucleotide cGAMP, which in turn activates the adaptor protein stimulator of interferon genes (STING) [4]. Subsequently, STING dimerizes and translocates from the endoplasmatic reticulum (ER) to the Golgi, where it interacts with TANK-binding kinase 1 (TBK1) resulting in the phosphorylation of both STING and TBK1 [5–8]. Phosphorylated TBK1 then phosphorylates and thereby activates the transcription factor interferon regulatory factor 3 (IRF3) [9,10]. IRF3 activation results in its dimerization and translocation into the nucleus, where IRF3 associates with the transcriptional cofactor CREB-binding protein (CBP)/p300 [11–13]. Recruitment of IRF3 and CBP/p300, among other transcription factors and chromatin modifying proteins, to the IFN-β enhanceosome finally results in transcription of the type I IFN IFN-β [14–16]. RNA sensing by RIG-I as well as other RIG-I-like receptors (RLRs) also results in the activation of TBK1 and IRF3 and subsequent type I IFN transcription, mainly via the adaptor protein mitochondrial antiviral signaling protein (MAVS) [3,17,18]. After transcription and subsequent secretion of type I IFNs, including IFN-α and IFN-β, they bind to the IFN-α/β receptor (IFNAR), which activates a signaling cascade that ultimately leads to the assembly of the interferon-stimulated gene factor 3 (ISGF3) complex consisting of the signal transducer and activator of transcription 1 and 2 (STAT1, STAT2) and IRF9. After translocation into the nucleus, the ISGF3 complex binds to interferon-stimulated response elements (ISREs) within the promoter regions of ISGs thereby regulating their expression [19].

Alpha thalassemia/mental retardation syndrome X-linked (ATRX) belongs to the SWI/SNF family of chromatin remodeling proteins and is mainly localized at heterochromatin, such as pericentromeric heterochromatin and telomeres [20–23]. Within these regions, ATRX and its interaction partner death domain-associated protein 6 (Daxx) act as a chromatin remodeling complex that maintains heterochromatin structure by facilitating the incorporation of the histone variant H3.3 [24,25]. The *ATRX* gene has been originally identified because of its involvement in the ATRX syndrome, an X-linked mental retardation syndrome comprising severe psychomotor retardation, distinct facial features, genital anomalies and α-thalassemia. The ATRX syndrome is caused by constitutional or acquired mutations in the *ATRX* gene, resulting in decreased ATRX protein levels or activity [26,27]. Since then, ATRX has been characterized as an important transcriptional regulator. Studies have shown that ATRX is able to facilitate the transcription of α-globin by negatively regulating the incorporation of the histone macroH2A1 into chromatin [28]. Moreover, multiple studies have shown an association of ATRX and the histone variant H3.3 with guanine-rich tandem repeats. These sequences are predicted to form non-B DNA structures known as G-quadruplexes (G4s). Although the exact mechanism remains unclear, it has been suggested that ATRX binds to and resolves G4s by incorporating H3.3 thereby facilitating the transcription of genes close to these structures [22,29,30]. Besides its role as a transcriptional regulator, ATRX is known to act as a suppressor of the alternative lengthening of telomers (ALT) in cancer cells. In a comprehensive study, it has been shown that 90% of *in vitro* immortalized ALT cell lines display a loss of ATRX [31]. Additionally, several studies have indicated that the role of ATRX in the deposition of H3.3 and its association with G4s contribute to its activity as an ALT suppressor [32,33].

ATRX is not only associated with heterochromatin but has also been found to localize at promyelocytic leukemia nuclear bodies (PML-NBs), together with Daxx [34]. PML-NBs are dynamic organelles in the nucleus that are formed by the key component PML as well as multiple other proteins and play an important role in intrinsic immunity against viral infections [35]. In particular, ATRX together with Daxx has been described to efficiently repress viral gene expression of numerous viruses including human cytomegalovirus (HCMV), herpes simplex virus type 1 (HSV-1) and adenovirus type 5 (Ad5) [36–39]. In turn, these viruses have developed diverse strategies to counteract the antiviral activity of PML-NBs. One of the strategies involves to directly target ATRX utilizing different mechanisms. During HCMV and Epstein-Barr virus (EBV) infection, ATRX is displaced from PML-NBs by the disruption of its interaction with Daxx, which is mediated by the viral regulatory proteins pp71 and BNRF1, respectively [37,40]. In contrast, the tegument protein ORF75 of Kaposi’s sarcoma-associated herpesvirus (KSHV) induces the degradation of ATRX [41]. Intriguingly, recent results from our group and others have revealed that PML-NBs, especially the key component PML, not only act as restriction factors but also play a role in innate immune signaling [42–45]. Moreover, another histone H3.3 chaperone, histone cell cycle regulator (HIRA), that has been shown to localize to PML-NBs upon herpesvirus infection as well as IFN stimulation has recently also been identified to contribute to both intrinsic and innate immune responses [46–48].

We therefore wanted to specifically investigate the role of ATRX as a novel regulator of the innate immune system. In this study, we show that the depletion of ATRX results in a suppressed activation of DNA and RNA sensing pathways, which in turn leads to decreased IFN induction and thereby an overall poor antiviral state in these cells. Moreover, we were able to show that ATRX interacts with IRF3 and is associated with the IFN-β promoter. Whole transcriptome sequencing revealed that ATRX specifically regulates the transcription of a distinct set of ISGs and thereby further contributes to the innate immune response. Utilizing ATAC-seq, we could also show that ATRX enhances chromatin accessibility specifically upon IFN signaling emphasizing its critical role in the IFN response. Moreover, chromatin immunoprecipitation (ChIP) analysis revealed an association of ATRX at responsive genes, identifying a potential mechanism of ATRX-mediated transcriptional regulation. Overall, our results led us to the conclusion that ATRX acts as a novel co-regulator of the innate immune system and viral antagonization of ATRX consequently represents an efficient strategy to interfere with both intrinsic and innate immune mechanisms.

## Results

### Depletion of ATRX impairs the innate immune response to HCMV infection

**Fig 1:**
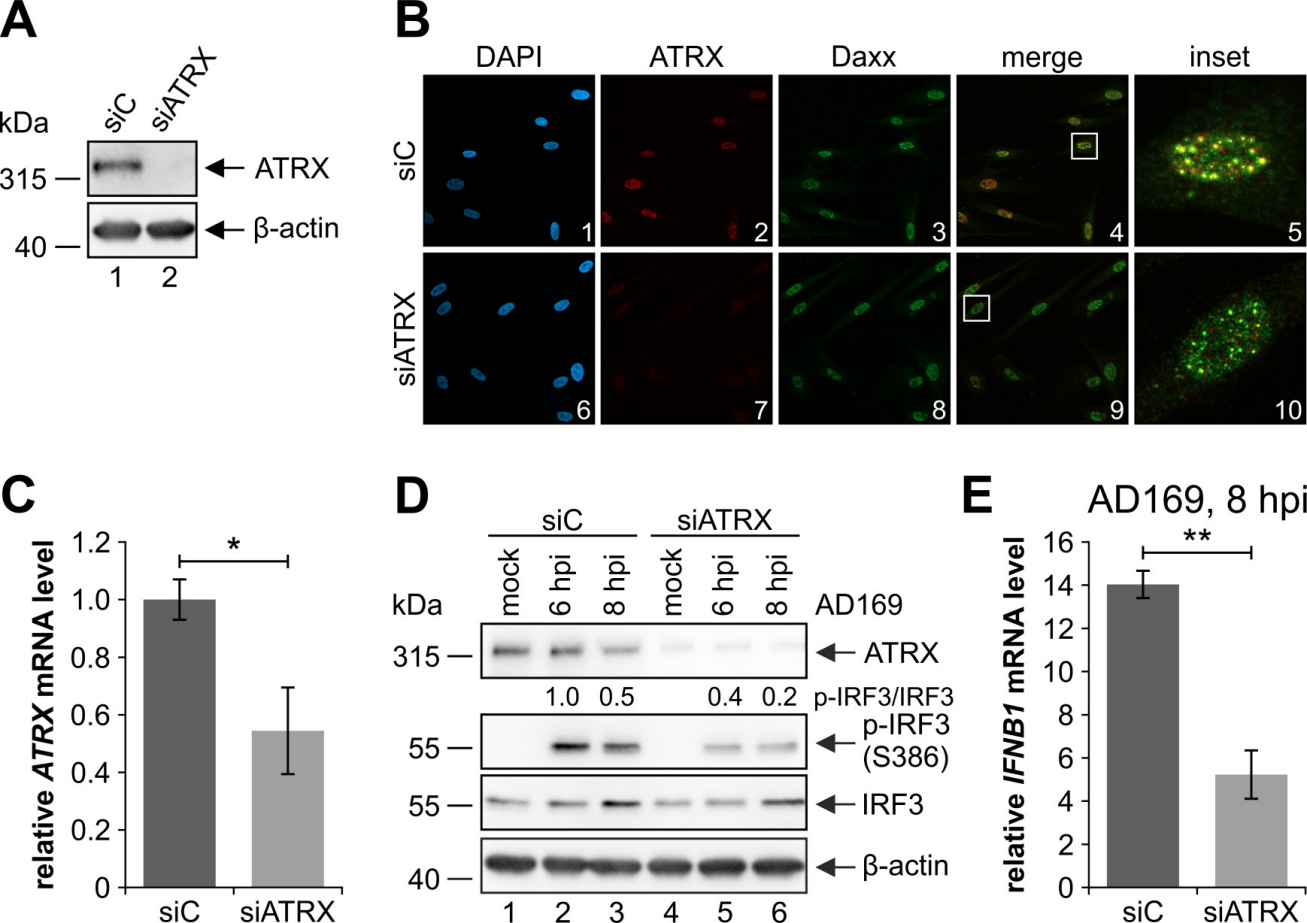
Suppressed innate immune response to HCMV infection in ATRX knockdown HFFs. (A) ATRX knockdown efficiency in siATRX cells was assessed by Western blotting; β-actin served as an internal loading control. (B) For further characterization of the siATRX cells, ATRX and its interaction partner Daxx were immunostained. 4’,6-diamidino-2-phenylindole (DAPI) was counterstained to visualize cellular nuclei. (C) To determine *ATRX* mRNA levels, total RNAs were isolated from ATRX knockdown HFFs (siATRX) and respective control HFFs (siC) and RT-qPCR was performed. (D+E) ATRX knockdown HFFs and respective control HFFs were either mock-infected or infected with the laboratory HCMV strain AD169 (MOI of 1). (D) At 6 and 8 hpi, cells were harvested for Western blot analyses to determine IRF3 phosphorylation; β-actin served as an internal loading control. Signal intensities were quantified relative to infected siC cells at 6 hpi (lane 2). (E) Additionally, total RNAs were isolated at 8 hpi and RT-qPCR was performed to determine *IFNB1* induction. (C+E) Depicted values were calculated from triplicates relative to (C) untreated or (E) mock-infected siC cells using *GAPDH* as a housekeeping gene and are shown as mean ± SD. One out of (C) four or (E) two independent experiments is shown. Statistical analysis was performed with respective ΔCq-values using a student’s *t*-test (unpaired, two-tailed); *p<0.05, **p<0.01. (A+B+D) The following antibodies were used: (A+D) anti-ATRX (39-f), anti-β-actin (AC-15); (D) anti-phospho-IRF3 (Ser386) (EPR2346), anti-IRF3 (D6I4C); (B) anti-ATRX (D-5), anti-Daxx (M112).

It has previously been shown that ATRX is involved in the activation of the cGAS-STING DNA sensing pathway in cancer cells that use the ALT pathway to maintain telomere length [49]. As the cGAS-STING DNA sensing pathway is crucial for the initial cellular response to DNA viruses, we wanted to further characterize the role of ATRX in the DNA sensing pathway during HCMV infection. To investigate this, we first generated HFFs with a stable shRNA-mediated knockdown of ATRX (siATRX) and respective control HFFs (siC) using lentiviral gene transfer. The siATRX cells displayed a significant reduction of ATRX expression both on the protein and mRNA level (Fig 1A–1C). Immunofluorescence analyses demonstrated a selective loss of ATRX expression (Fig 1B, panel 7), whereas expression of its interaction partner Daxx remained unchanged (Fig 1B, panel 8). After having confirmed the knockdown efficiency of the siATRX cells, we investigated the effect of ATRX on the DNA sensing pathway in the context of infection. For this, we infected siATRX and siC cells with HCMV strain AD169 at a multiplicity of infection (MOI) of 1. At 6 and 8 hpi, cells were harvested and subjected to Western blot analyses to examine the phosphorylation of IRF3 at serine 386 (Fig 1D). Phosphorylation at this residue has been described as a key step for signal transduction upon cytosolic DNA sensing [11,50]. As expected, phosphorylation of IRF3 at serine 386 was induced upon infection of the control cells at both analyzed time points (Fig 1D, lanes 2 and 3). However, in ATRX knockdown HFFs phosphorylation of IRF3 was decreased by up to 60%, indicating a suppressed activation of the DNA sensing pathway (Fig 1D, lanes 5 and 6). As the type I IFN IFN-β is induced by IRF3 after its activation [11], we next investigated the effect of the ATRX knockdown on the transcription of *IFNB1* via quantification of *IFNB1* mRNA levels by RT-qPCR (Fig 1E). *IFNB1* mRNA levels were significantly reduced by approximately 63% in siATRX cells compared to siC cells, which is consistent with the effect of the ATRX knockdown on IRF3 phosphorylation. Taken together, these results show that depletion of ATRX suppresses the innate immune response to HCMV infection suggesting a role of ATRX in the cytosolic DNA sensing pathway.

### ATRX regulates the cytosolic cGAS-STING DNA sensing pathway in a cGAS-independent manner

**Fig 2:**
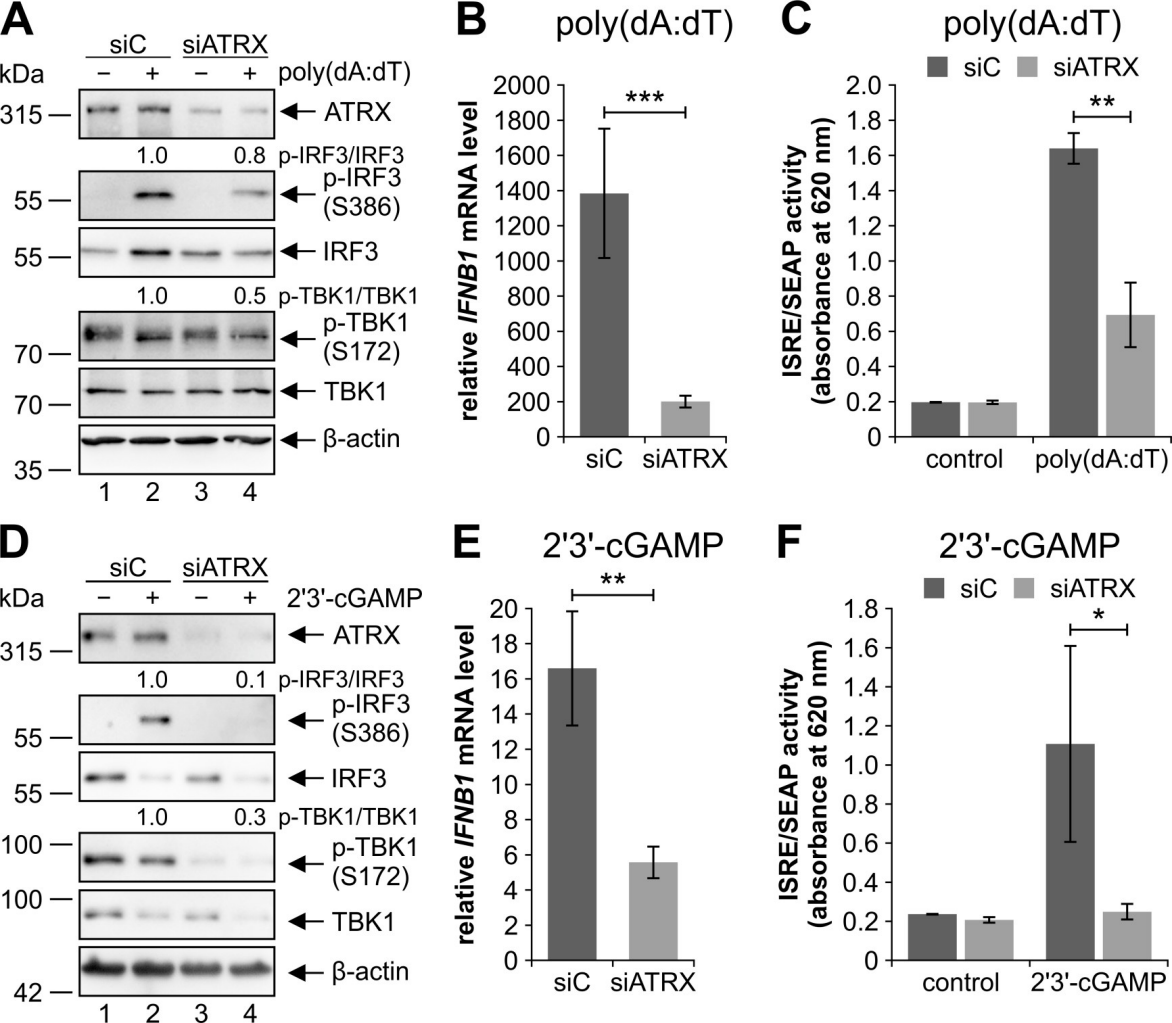
Repression of the cGAS-STING DNA sensing pathway in ATRX knockdown HFFs. ATRX knockdown HFFs (siATRX) and respective control cells (siC) were treated with (A-C) 0.1 μg/ml poly(dA:dT) and (B+C) DMSO or (D-F) 50 μg/ml 2’3’-cGAMP for 24 h. (A+D) Cells were harvested for Western blot analyses to determine the phosphorylation of IRF3 and TBK1; β-actin served as an internal loading control. Signal intensities were quantified relative to treated siC cells (lane 2). The following antibodies were used: anti-phospho-IRF3 (Ser386) (EPR2346), anti-IRF3 (D6I4C), anti-phospho-TBK1 (Ser172) (D52C2), anti-TBK1 (D1B4), anti-β-actin (AC-15); (A) anti-ATRX (39-f); (D) anti-ATRX (HPA001906). (B+E) Total RNAs were isolated and RT-qPCR was performed to determine *IFNB1* induction. Depicted values were calculated from triplicates relative to untreated siC cells using *GAPDH* as a housekeeping gene and are shown as mean ± SD. One out of (B) three or (E) two independent experiments is shown. Statistical analysis was performed with respective ΔCq-values using a student’s *t*-test (unpaired, two-tailed); **p<0.01, ***p<0.001. (C+F) Supernatants of stimulated cells were harvested and mixed with HEK-Blue IFN-α/β cells. Type I IFNs in the supernatants were quantitated by measuring SEAP activity produced by HEK-Blue IFN-α/β cells using QUANTI-Blue. Depicted values are derived from triplicates and are shown as mean ± SD. Statistical analysis was performed using a student’s *t*-test (unpaired, two-tailed); *p<0.05, **p<0.01.

We next wanted to exclude that diminished innate immune responses after HCMV infection are due to the activity of ATRX as an antiviral restriction factor and additionally prove a direct effect of ATRX on the cGAS-STING DNA sensing pathway [37]. Therefore, we stimulated ATRX knockdown HFFs with 0.1 μg/ml poly(dA:dT), a synthetic double-stranded DNA sequence that is recognized by different DNA sensors triggering the type I IFN production (Fig 2A–2C). Additionally, we investigated phosphorylation of the kinase responsible for IRF3 activation, TBK1, which is phosphorylated at serine 172 in its activation loop [9]. Like IRF3, phosphorylation of TBK1 could be detected after stimulation with poly(dA:dT) (Fig 2A, lane 2). In ATRX knockdown HFFs, both IRF3 and TBK1 phosphorylation were reduced by 20% and 50%, respectively (Fig 2A, compare lanes 2 and 4). Quantification of independent experiments revealed that the phosphorylation of IRF3 and TBK1 relative to total protein levels was significantly reduced in ATRX knockdown HFFs (S1A and S1B Fig). Consistent with results obtained in Western blots, stimulation of ATRX knockdown HFFs with poly(dA:dT) resulted in significantly reduced *IFNB1* transcription by approximately 86% compared to control cells (Fig 2B). We next wanted to determine whether reduced *IFNB1* mRNA levels in ATRX knockdown HFFs result in decreased IFN-β secretion. For this, we determined total type I IFN levels in the supernatant of poly(dA:dT)-stimulated ATRX knockdown cells using HEK-Blue IFN-α/β cells as a reporter cell line for human type I IFN (Fig 2C). These cells induce the production of a secreted embryonic alkaline phosphatase (SEAP) in the presence of IFN-α or IFN-β. As illustrated in Fig 2C, SEAP activity and consequently secretion of type I IFN was significantly reduced after depletion of ATRX.

Having shown that depletion of ATRX affects the phosphorylation of TBK1 and IRF3, we investigated whether ATRX acts upstream of these factors. Prior to phosphorylation of TBK1 and IRF3, STING gets activated by 2’3’-cGAMP, which is produced by cGAS upon DNA recognition [2,51]. In order to investigate whether ATRX acts independent of the DNA sensor cGAS, we treated the ATRX knockdown HFFs directly with the STING ligand 2’3’-cGAMP for 24 h (Fig 2D–2F). As expected, 2’3’-cGAMP induced phosphorylation of IRF3 and TBK1 in control cells, whereas phosphorylation of both proteins was reduced in ATRX knockdown cells by 90% and 70%, respectively (Fig 2D, compare lane 2 and 4). Quantification of independent experiments confirmed a significant reduction of IRF3 and TBK1 phosphorylation relative to total protein levels in ATRX knockdown HFFs (S1C and S1D Fig). Although 2’3’-cGAMP (50 μg/ml) had a less pronounced effect on the activation of *IFNB1* transcription compared to poly(dA:dT), we still observed significantly reduced *IFNB1* transcription as well as type I IFN secretion in siATRX cells (Fig 2E and 2F). These results indicate an impaired activation of the cGAS-STING DNA sensing pathway in ATRX-depleted HFFs not only after poly(dA:dT) stimulation but also after treatment with the STING ligand 2’3’-cGAMP. Thus, we concluded that ATRX most likely affects the cytosolic cGAS-STING DNA sensing pathway downstream of the initial activation step by cGAS.

### ATRX interacts with IRF3 and positively affects IRF3-mediated ISG induction

**Fig 3:**
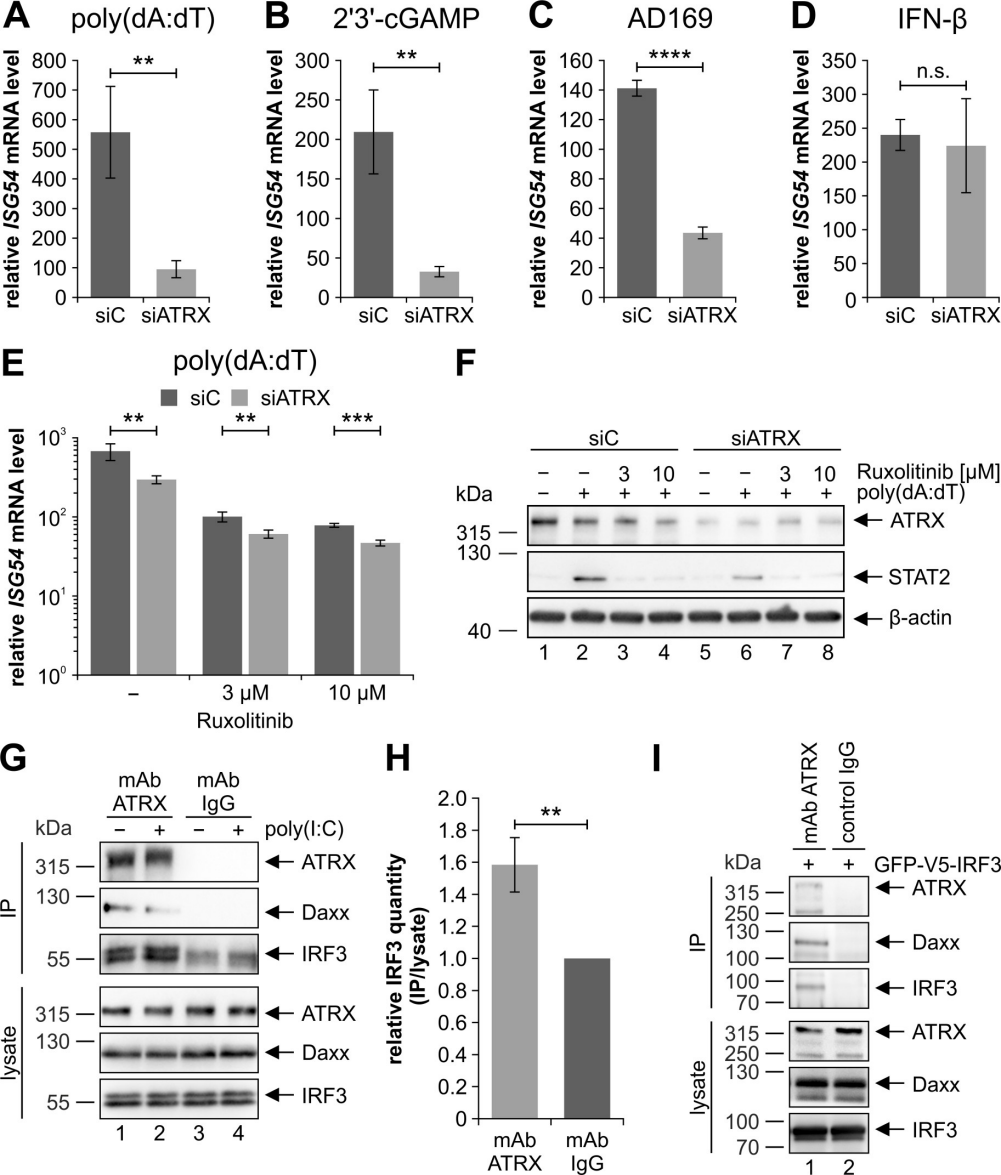
ATRX interacts with IRF3 and modulates IRF3 activity. Stable ATRX knockdown HFFs (siATRX) and respective control HFFs (siC) were (A) treated with DMSO and 0.1 μg/ml poly(dA:dT) for 24 h, (B) treated with 50 μg/ml 2’3’-cGAMP for 24 h, (C) infected with the laboratory HCMV strain AD169 (MOI of 1) for 8 h or (D) treated with 3.28 × 10^3^ U/ml IFN-β for 24 h. (E+F) Alternatively, cells were treated with 0.1 μg/ml poly(dA:dT) and 3 μM or 10 μM Ruxolitinib for 24 h. (A-E) Total RNAs were prepared and RT-qPCR was performed to determine transcription of the IRF3-responsive gene *ISG54*. Depicted values were calculated from triplicates relative to untreated siC cells using *ACTB* or *GAPDH* as housekeeping genes and are shown as mean ± SD. One out of at least two independent experiments is shown. Statistical analysis was performed with respective ΔCq-values using a student’s *t*-test (unpaired, two-tailed); n.s.: not significant, **p<0.01, ***p<0.001, ****p<0.0001. (F) Cells were harvested for Western Blot analyses to determine STAT2 expression; β-actin served as internal loading control. The following antibodies were used: anti-ATRX (HPA001906), anti-STAT2 (A7), anti-β-actin (AC-15). (G-I) HEK293T cells were (G) stimulated with 0.5 μg/ml poly(I:C) or (I) transfected with a plasmid coding for GFP-V5-IRF3. After 24 h, cells were immunoprecipitated with an ATRX antibody (D1N2E) or an IgG isotype control antibody. The following antibodies were used for detection of proteins in the immunoprecipitate (IP) and in the whole cell lysate (lysate): ATRX (HPA001906), Daxx (E94) and IRF3 (D6I4C). **(H)** Quantification of co-immunoprecipitated IRF3 relative to total IRF3 levels in the lysate control. Signal intensities were calculated relative to the IgG isotype control. Data were obtained from four independent experiments and are shown as mean ± SD. Statistical analysis was performed using a student’s *t*-test (one sample, two-tailed); **p<0.01.

Next, we wanted to investigate whether the decreased *IFNB1* mRNA levels in ATRX knockdown HFFs display a direct effect of ATRX on IRF3 activity. Prior to that, we ensured that total protein levels as well as mRNA levels of IRF3 are not affected by the ATRX knockdown (S2 Fig). The activity of the transcription factor IRF3 is not limited to facilitating the transcription of *IFNB1*. In fact, there are several reports describing that IRF3 directly regulates the transcription of different immunoregulatory genes (e.g. *CCL5*, *ISG54*, *ISG56*) [52–55]. Therefore, we analyzed the transcription of *ISG54* as an IRF3-responsive gene in ATRX knockdown HFFs upon stimulation with different agents (Fig 3A–3D). We treated ATRX knockdown HFFs and respective control cells with poly(dA:dT) (Fig 3A) and 2’3’-cGAMP (Fig 3B) as direct stimulants of the cGAS-STING DNA sensing pathway. As expected, poly(dA:dT) and 2’3’-cGAMP induced transcription of *ISG54*, however, poly(dA:dT) treatment showed the stronger induction of approximately 558-fold in control cells (Fig 3A) compared to 209-fold induction upon 2’3’-cGAMP treatment (Fig 3B). In ATRX-depleted cells, treatment with poly(dA:dT) and 2’3’-cGAMP led to a significant reduction of *ISG54* mRNA levels by approximately 83% and 84%, respectively (Fig 3A and 3B). Infection with AD169 also induced *ISG54* transcription approximately 141-fold in control cells, whereas ATRX-depleted cells displayed a reduced induction of *ISG54* mRNA levels by approximately 69% (Fig 3C). In order to exclude an effect of ATRX on *ISG54* transcription via IFN secretion, we additionally treated the cells with IFN-β for 24 h (Fig 3D). IFN-β activates *ISG54* transcription via the JAK-STAT signaling pathway and therefore in an IRF3-independent manner. Intriguingly, upon stimulation with IFN-β, the induction of *ISG54* mRNA levels was not altered in ATRX-depleted cells compared to control cells (Fig 3D). These results suggest that ATRX regulates IRF3 activity independent of IFN secretion. To further corroborate this, we additionally treated ATRX knockdown cells with the JAK1 and JAK2 inhibitor Ruxolitinib, which should prevent type I IFN-induced *ISG54* transcription (Fig 3E and 3F) [56]. Treatment with poly(dA:dT) alone induced *ISG54* mRNA levels approximately 650-fold, whereas *ISG54* mRNA levels were induced approximately 97-fold and 75-fold after parallel treatment with poly(dA:dT) and 3 μM or 10 μM Ruxolitinib, respectively (Fig 3E). This indicates that Ruxolitinib treatment was successful and IFN-induced *ISG54* transcription was inhibited. Interestingly, *ISG54* mRNA levels were still significantly reduced in ATRX knockdown HFFs after treatment with both poly(dA:dT) and Ruxolitinib, suggesting that ATRX indeed regulates IRF3 activity independent of the JAK-STAT signaling pathway. We additionally confirmed successful Ruxolitinib treatment by Western blotting (Fig 3F). In control cells, Ruxolitinib and poly(dA:dT) treatment showed STAT2 protein levels comparable to untreated cells (Fig 3F, lanes 1, 3 and 4). This was observed for both applied Ruxolitinib concentrations. Furthermore, the Western blot revealed reduced STAT2 protein levels in poly(dA:dT)-treated ATRX knockdown HFFs compared to the control cells (Fig 3F, compare lanes 2 and 6). This is consistent with the reduced secretion of type I IFNs in these cells as shown before (Fig 2C).

Next, we asked whether ATRX could interact with IRF3. To analyze this, we stimulated HEK293T cells with 0.5 μg/ml poly(I:C) high molecular weight (HMW) for 24 h and immunoprecipitated endogenous ATRX using an ATRX specific antibody (Fig 3G). Precipitation with an IgG isotype control served as negative control. Indeed, endogenous IRF3 co-immunoprecipitated with ATRX, which occurred independent of poly(I:C) HMW stimulation (Fig 3G, lanes 1 and 2). Likewise, Daxx, which served as a positive control, specifically co-immunoprecipitated with ATRX (Fig 3G, lanes 1 and 2). In the case of IRF3, less abundant signals could also be detected in the IgG control, which are most likely ascribed to the IgG heavy chain (Fig 3G, lanes 3 and 4). Quantification of independent experiments revealed a significant enrichment of the IRF3 signal by approximately 60% upon precipitation of ATRX (Fig 3H). To circumvent the problem of the obscuring IgG heavy chain signal and thus ensure specific co-precipitation of IRF3, we additionally transfected HEK293T cells with a GFP- and V5-tagged IRF3 and immunoprecipitated again endogenous ATRX. In this experimental setting, a specific co-immunoprecipitation of endogenous Daxx as well as the transfected IRF3 construct was observed only when ATRX was precipitated (Fig 3I, compare lanes 1 and 2). Taken together, these results further imply a regulatory role of ATRX in the innate immune system and additionally suggest that ATRX interacts with IRF3 and thereby potentially affects IRF3-mediated transcriptional regulation.

### ATRX regulates the RIG-I/MDA-5 signaling pathway

**Fig 4:**
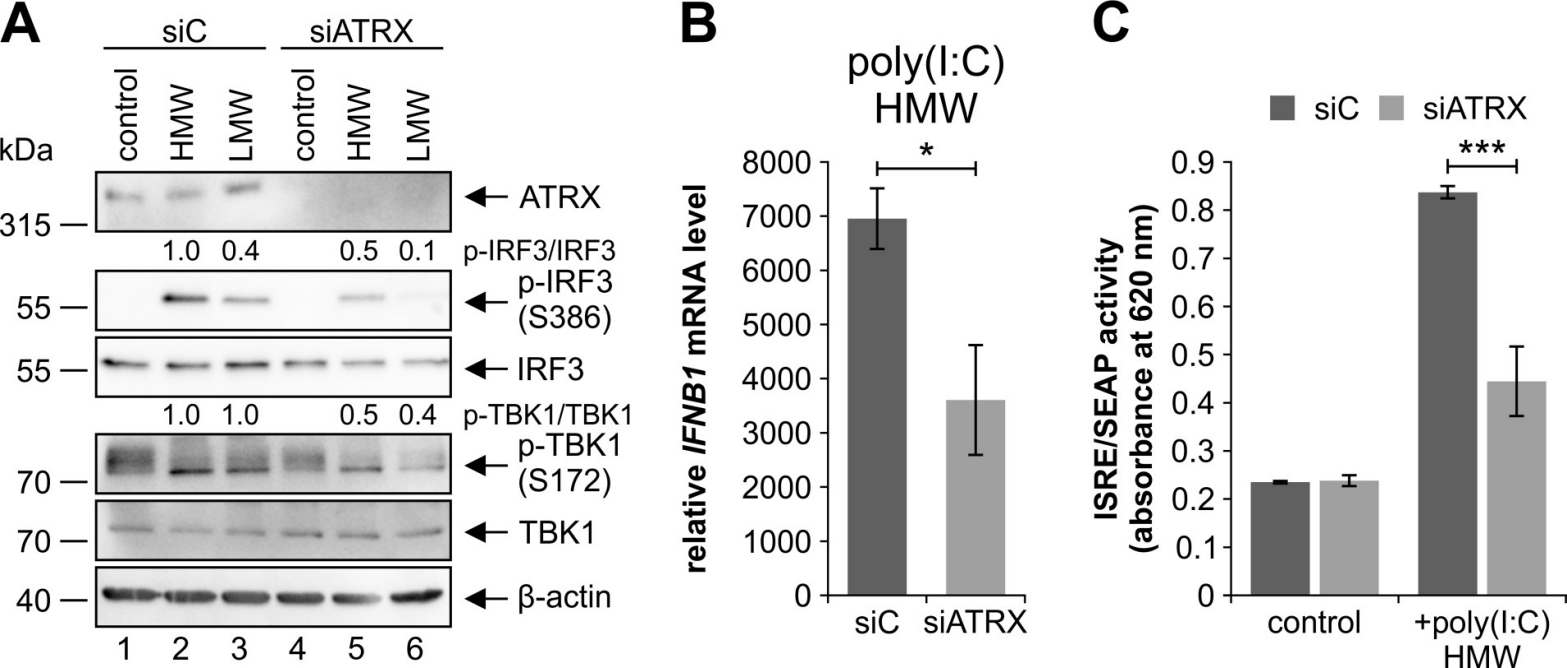
Repression of the RIG-I/MDA-5 signaling pathway in ATRX knockdown HFFs. Stable ATRX knockdown HFFs (siATRX) and respective control cells (siC) were treated with (A) 0.5 μg/ml poly(I:C) HMW and poly(I:C) LMW, (B) 0.5 μg/ml poly(I:C) HMW or (C) 0.1 μg/ml poly(I:C) HMW for 24 h. (A) Cells were harvested for Western blotting to determine the phosphorylation of IRF3 and TBK1; β-actin served as internal loading control. Signal intensities were quantified relative to poly(I:C) HMW-treated siC cells (lane 2). The following antibodies were used: anti-ATRX (39-f), anti-phospho-IRF3 (Ser386) (EPR2346), anti-IRF3 (D6I4C), anti-phospho-TBK1 (Ser172) (D52C2), anti-TBK1 (D1B4), anti-β-actin (AC-15). (B) Total RNAs were prepared and RT-qPCR was performed to determine *IFNB1* induction. Depicted values were calculated from triplicates relative to untreated siC cells using *GAPDH* as a housekeeping gene and are shown as mean ± SD. One out of two independent experiments is shown. Statistical analysis was performed with respective ΔCq-values using a student’s *t*-test (unpaired, two-tailed); *p<0.05. (C) Supernatants were harvested and mixed with HEK-Blue IFN-α/β cells. Type I IFNs in the supernatants were quantitated by measuring SEAP activity produced by HEK-Blue IFN-α/β cells using QUANTI-Blue. Depicted values were derived from triplicates and are shown as mean ± SD. Statistical analysis was performed using a student’s *t*-test (unpaired, two-tailed); ***p<0.001.

Activation of the kinase TBK1 followed by IRF3 activation and subsequent *IFNB1* and *ISG54* transcription is not only mediated by cGAS and STING upon DNA sensing, but also via sensing of RNA by RLRs [3,17]. Therefore, we presumed that ATRX might also be a regulator of the RNA sensing pathway. In order to confirm this, we treated ATRX knockdown HFFs and respective control cells with 0.5 μg/ml poly(I:C) high molecular weight (HMW) or poly(I:C) low molecular weight (LMW), which are synthetic dsRNA sequences that are both recognized by RIG-I/MDA-5 (Fig 4). Both poly(I:C) HMW and LMW induced phosphorylation of IRF3 at serine 386 and TBK1 at serine 172 in control cells (Fig 4A, lanes 2 and 3). However, poly(I:C) HMW appeared to activate the RNA sensing pathway to a greater extent than poly(I:C) LMW as visible by the stronger phosphorylation of IRF3. Nonetheless, phosphorylation of IRF3 and TBK1 was reduced in ATRX knockdown HFFs compared to control cells after treatment with poly(I:C) HMW as well as poly(I:C) LMW, indicating an impaired activation of the RNA sensing pathway (Fig 4A, compare lanes 2, 3 and 5, 6). Quantification of independent experiments revealed that the phosphorylation of IRF3 and TBK1 relative to total protein levels was significantly reduced in poly(I:C) HMW-treated ATRX knockdown HFFs (S1E and S1F Fig). As poly(I:C) HMW appeared to have the stronger effect on RIG-I activation, we decided to carry out subsequent experiments with poly(I:C) HMW. In ATRX knockdown cells transcription of *IFNB1* was significantly reduced upon stimulation with poly(I:C) HMW by 48% (Fig 4B). As expected, this reduced *IFNB1* transcription also led to a significant decrease of the secretion of type I IFNs, as shown by reduced SEAP activity (Fig 4C). From these results, we concluded that ATRX not only acts as a regulator of the cGAS-STING DNA sensing pathway but also plays a crucial role in RIG-I-dependent RNA sensing further underlining that ATRX has emerged as an important co-regulator of the initial innate immune response.

### ATRX positively regulates type I and type II IFN signaling

**Fig 5:**
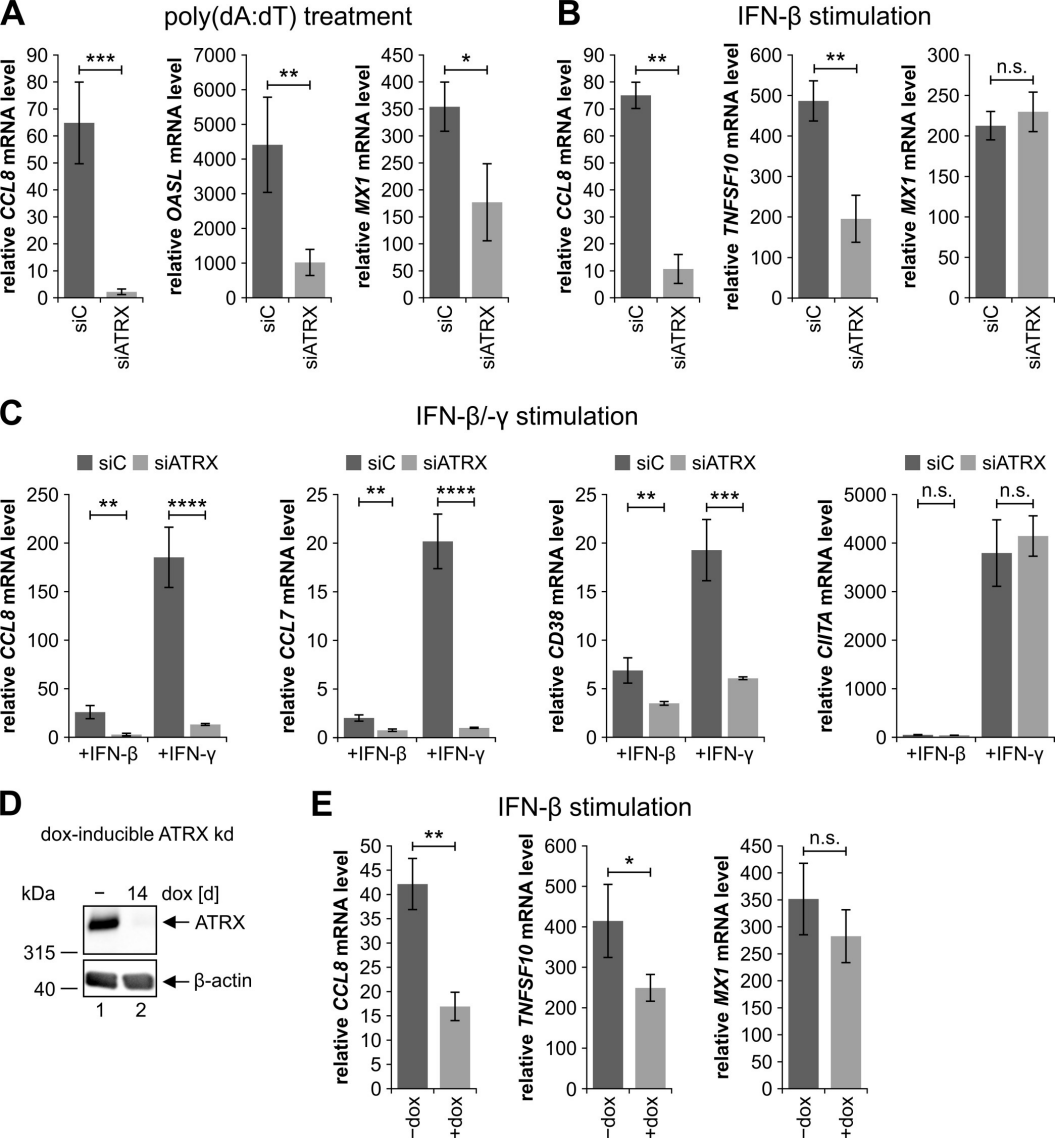
Impaired ISG expression in ATRX knockdown HFFs. (A-C) ATRX knockdown HFFs (siATRX) and respective control cells (siC) were treated with (A) 0.1 μg/ml poly(dA:dT), (B) IFN-β (3.28 to 3.75× 10^3^ U/ml), or (C) IFN-β (3.87 × 10^3^ U/ml) and IFN-γ (0.2 μg/ml) for 24 h. Subsequently, total RNAs were prepared and RT-qPCR was performed to determine transcription of the ISGs (A) *CCL8*, *OASL* and *MX1*, (B) *CCL8*, *TNFSF10* and *MX1* or (C) *CCL8*, *CCL7*, *CD38* and *CIITA*. (D) HFFs with a doxycycline-inducible expression of an shRNA targeting ATRX were either left untreated (−) or treated with 500 ng/ml doxycycline (dox) for 14 days (d). Subsequently, cells were harvested for Western blot analyses to determine the knockdown efficiency; β-actin served as an internal loading control. The following antibodies were used: anti-ATRX (39-f), anti-β-actin (AC-15). kd: knockdown (E) Doxycycline-inducible ATRX knockdown HFFs were either left untreated (−dox) or treated with 500 ng/ml dox for 14 d to induce an ATRX knockdown, followed by stimulation with IFN-β (3.75 × 10^3^ U/ml) for 24 h. Subsequently, total RNAs were prepared and RT-qPCR was performed to determine transcription of the ISGs *CCL8*, *TNFSF10* and *MX1*. (A+B+C+E) Depicted values were calculated from triplicates relative to untreated control cells using *GAPDH* as a housekeeping gene and are shown as mean ± SD. One out of at least two independent experiments is shown. Statistical analysis was performed with respective ΔCq-values using a student’s *t*-test (unpaired, two-tailed); n.s.: not significant, *p<0.05, **p<0.01, ***p<0.001, ****p<0.0001.

As ATRX knockdown HFFs displayed an impaired secretion of type I IFNs after activation of DNA and RNA sensing pathways, we speculated that this also results in a reduced transcription of different ISGs. To confirm our hypothesis, we investigated mRNA levels of the ISGs *CCL8*, *OASL* and *MX1* in ATRX knockdown HFFs after activation of the DNA sensing pathway by poly(dA:dT) (Fig 5A). As expected, the reduced secretion of type I IFNs in ATRX knockdown HFFs after stimulation with poly(dA:dT) resulted in a reduced expression of the different ISGs. The induction of *CCL8* expression was reduced the most by 97%, while the induction of *OASL* and *MX1* was reduced by 77% and 50%, respectively. These results further highlight the important role of ATRX in establishing an antiviral state upon activation of the DNA sensing pathway.

So far, our results have suggested that ATRX indirectly regulates ISG expression by positively regulating IRF3 activity and thus type I IFN production. As a next step, we were eager to investigate a potential direct effect of ATRX on ISG expression, especially because PML, the key component of PML-NBs, has been implicated as a positive regulator of type I and type II IFN signaling [42–45]. In order to investigate whether ATRX is involved in regulating ISG expression directly, we stimulated ATRX knockdown HFFs and respective control cells with IFN-β (Fig 5B). Transcription of all investigated ISGs (*CCL8*, *TNFSF10*, *MX1*) was efficiently induced in control cells upon stimulation with IFN-β, although it should be noted that *CCL8* mRNA levels were only slightly induced upon IFN-β stimulation (75-fold) compared to *TNFSF10* and *MX1* mRNA levels (486-fold and 213-fold, respectively) (Fig 5B). In ATRX knockdown HFFs, *CCL8* and *TNFSF10* mRNA levels were significantly reduced by 86% and 60%, respectively. Remarkably, *MX1* mRNA levels were not altered in ATRX knockdown HFFs, suggesting that the expression of not all but a limited set of ISGs is regulated by ATRX. This is in accordance with our previous results showing that ATRX depletion had no effect on *ISG54* transcription upon stimulation with IFN-β (Fig 3D). Surprisingly, the effects on ISG expression in ATRX knockdown HFFs are clearly distinctive from the effects observed in PML and Daxx knockdown HFFs (S3 Fig). In particular, we observed no reduced expression of any investigated ISG in Daxx knockdown HFFs, suggesting that ATRX does not require Daxx for regulating ISG expression. Moreover, *ISG54* and *MX1* mRNA levels were significantly reduced in PML knockdown HFFs, whereas no effects were detectable in ATRX knockdown HFFs (S3 Fig). These results further suggest a specific mode of action of ATRX in regulating expression of only a distinct set of ISGs.

Unlike *TNFSF10* and *MX1*, which are primarily induced by type I IFNs, *CCL8* expression is predominantly induced by the type II IFN IFN-γ [57]. This explains why *CCL8* shows only a mild induction after IFN-β stimulation compared to *TNFSF10* and *MX1*. Therefore, we decided to additionally investigate transcription of different ISGs, including *CCL8*, in ATRX knockdown HFFs upon stimulation with IFN-γ (Fig 5C). Analogous to the experiment before, total RNAs were isolated 24 h after stimulation with IFN-β or IFN-γ, followed by RT-qPCR to determine transcription of *CCL8*, *CCL7*, *CD38* and *CIITA* (Fig 5C). All investigated ISGs were primarily induced by IFN-γ, but also showed a slight induction upon stimulation with IFN-β (Fig 5C). Similar to the results from Fig 5B, *CCL8* mRNA levels were significantly reduced by approximately 89% in ATRX knockdown HFFs after stimulation with IFN-β. Intriguingly, this effect became even stronger after stimulation with IFN-γ (93% reduction). Moreover, *CCL7* and *CD38* mRNA levels were significantly reduced in ATRX knockdown HFFs after stimulation with either IFN-β or IFN-γ, while the effect was again more prominent upon IFN-γ stimulation (95% and 68% reduction, respectively) compared to IFN-β stimulation (62% and 49% reduction, respectively). *CIITA* displayed the highest induction upon IFN-γ stimulation (3794-fold) and only a mild induction upon IFN-β stimulation (49-fold). However, *CIITA* mRNA levels were not altered in ATRX knockdown HFFs, additionally suggesting that ATRX specifically regulates the expression of certain ISGs induced by type I and II IFNs.

In order to validate the phenomenon that not all investigated ISGs display reduced mRNA levels in the absence of ATRX, we generated HFFs with a doxycycline-inducible expression of an shRNA targeting ATRX using lentiviral gene transfer. In these cells, ATRX expression is unaltered and a knockdown is only induced upon addition of doxycycline (dox). Therefore, any potential indirect effects arising from culturing HFFs lacking ATRX expression should be diminished. As visible in Fig 5D, an efficient knockdown of ATRX was achieved after 14 days of dox treatment. Therefore, for all further experiments, inducible ATRX knockdown HFFs were treated with dox for 14 days or left untreated as a control. After assessing knockdown efficiency, we investigated the effect of the inducible ATRX knockdown on ISG expression (Fig 5E). Consistent with the results of Fig 5B, all ISGs were considerably induced by IFN-β to varying extents. Moreover, in dox-treated cells both *CCL8* and *TNFSF10* mRNA levels were significantly reduced by approximately 60% and 40%, respectively, whereas *MX1* mRNA levels were not different (Fig 5E). Consequently, this experiment confirms our prior results with stable ATRX knockdown HFFs. Taken together, these results show that ATRX positively regulates ISG expression by affecting IRF3-mediated production of type I IFNs and by regulating type I and type II IFN signaling.

### RNA-seq reveals that a distinct set of ISGs is regulated by ATRX

**Fig 6:**
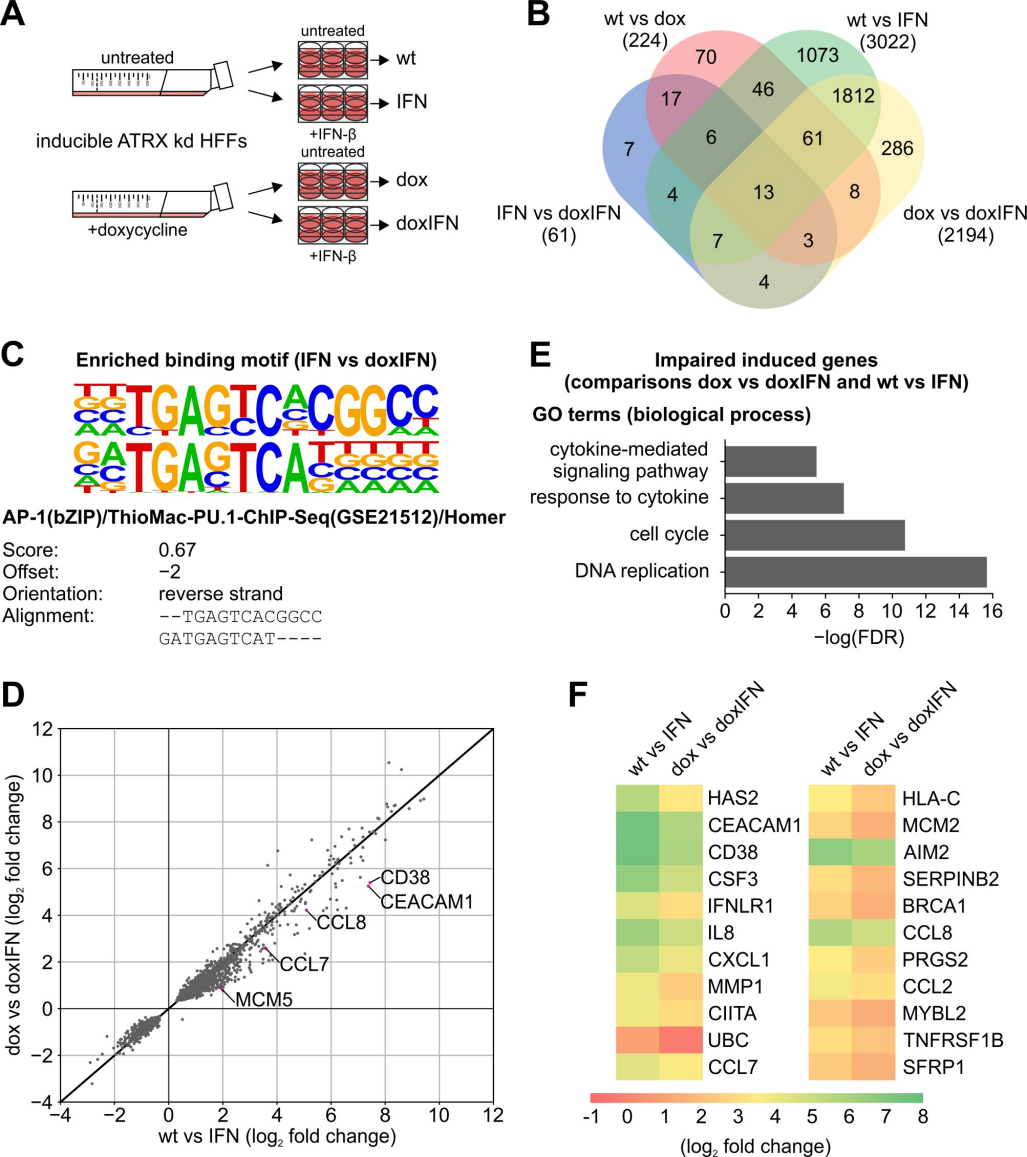
RNA-seq analysis of doxycycline-inducible ATRX knockdown HFFs stimulated with IFN-β. (A) Schematic representation of the experiment. Doxycycline-inducible ATRX knockdown (ATRX kd) HFFs were either treated with 500 ng/ml doxycycline (dox) for 14 days (d) or left untreated as a control, followed by stimulation with IFN-β (3.28 × 10^3^ U/ml) for 24 h. Subsequently, total RNAs were prepared and subjected to RNA-seq. Samples were divided in four groups of triplicates (untreated: wt; only IFN-β-treated: IFN; only dox-treated: dox; dox- and IFN-β-treated: doxIFN). (B) Venn-diagram of pairwise comparisons of the four groups. Differentially expressed genes were filtered to an adjusted p-value < 0.01. The following online tool was used: http://bioinformatics.psb.ugent.be/webtools/Venn/ (accessed 13 Sep 2021). (C) Homer *de novo* motif analysis of promoter regions of the differentially expressed genes from the comparison dox vs doxIFN. The top motif (p-value of 10^−14^) is shown (top) aligned with one of the matched known motifs (bottom). (D) Scatter plot of log_2_ fold changes from the comparison of dox vs doxIFN and wt vs IFN for every gene (n = 1893) with an adjusted p-value < 0.01. (E) Gene ontology (GO) terms of the category biological process for 85 corresponding proteins out of the top 100 hits with reduced log_2_ fold changes in dox vs doxIFN compared to wt vs IFN; FDR: false discovery rate. (F) Heat map of proteins belonging to the GO terms response to cytokine (GO:0034097) and cytokine-mediated signaling pathway (GO:0019221).

So far, we have investigated a limited set of ISGs using both stable and inducible ATRX knockdown HFFs. In order to get a better understanding of the role of ATRX in ISG transcription, we performed whole transcriptome sequencing (RNA-seq) using dox-inducible ATRX knockdown HFFs stimulated with IFN-β (Fig 6). Fig 6A summarizes the experimental strategy. As described earlier, the inducible ATRX knockdown HFFs were either treated with dox for 14 days or left untreated as a control. Afterwards, we stimulated the cells with IFN-β for 24 h or left the cells untreated followed by isolation of total RNAs, which were subjected to RNA-seq. Samples were divided in four groups of triplicates (untreated cells: wt; only IFN-β-treated cells: IFN; only dox-treated cells: dox; dox and IFN-β-treated cells: doxIFN) and differential expression analysis was performed. Using an adjusted p-value p < 0.01, we identified 3022 differentially expressed genes in control cells after treatment with IFN-β (wt vs IFN) and only 2194 differentially expressed genes in knockdown cells after treatment with IFN-β (dox vs doxIFN), whereas 1893 genes were in common between these two comparisons (Fig 6B). This indicates that significant changes regarding gene expression occur in ATRX-depleted cells following IFN-β stimulation. Additionally, 224 differentially expressed genes were identified in HFFs in which the ATRX knockdown was induced with dox but no IFN-β added (wt vs dox) and 61 differentially expressed genes were identified in control cells treated with interferon compared to knockdown cells treated with interferon (IFN vs doxIFN) (Fig 6B). First, we ensured that the knockdown of ATRX itself does not affect expression of genes related to innate immune signaling. For this, we analyzed the top 50 differentially expressed genes from the comparison wt vs dox (S1 Table). Notably, no genes were identified that could account for the observed effects of ATRX knockdown cells on IFN response as well as IRF3 signaling. Next, we sought to identify potential transcription factor binding sites in genes differentially regulated in ATRX-depleted cells upon IFN-β stimulation (IFN vs doxIFN), as these might reveal a general mechanism of ATRX-mediated gene regulation. For this, we analyzed the promoter regions of the 61 differentially expressed genes from the comparison IFN vs doxIFN using the Hypergeometric Optimization of Motif Enrichment (HOMER) analysis. The *de novo* motif analysis revealed an enrichment of a motif corresponding to the AP-1 motif (Fig 6C). This suggests that ATRX modulates the expression of AP-1 target genes upon IFN-β stimulation. As we were most interested in identifying novel genes regulated by ATRX upon stimulation with IFN-β, we next compared the log_2_ fold change (FC) of dox-induced ATRX knockdown cells treated with IFN-β (dox vs doxIFN) and respective control cells treated with IFN-β (wt vs IFN) and depicted the 1893 common genes in a scatter plot (Fig 6D). With this analysis, considerably more genes could be identified showing a reduced FC in ATRX knockdown cells (dox vs doxIFN) compared to control cells (wt vs IFN) and only a few genes with an increased FC. Among the genes with a reduced FC we also identified several ISGs from our previous RT-qPCR experiments (*CCL8*, *CCL7* and *CD83*). Additionally, we identified numerous novel genes with a reduced FC in dox vs doxIFN compared to wt vs IFN. The top 100 hits with a negative FC are listed in S2 Table. From these top 100 hits, 85 corresponding proteins (highlighted in black) were further analyzed using the STRING database and we identified a highly clustered network (S4 Fig) [58]. Functional analysis of these 85 proteins revealed enrichment of the gene ontology (GO) terms DNA replication (GO:0006260), cell cycle (GO:0007049), response to cytokine (GO:0034097) and cytokine-mediated signaling pathway (GO:0019221) among others (Fig 6E). As we aimed to identify novel ISGs potentially regulated by ATRX, we focused on the genes sorted according to the GO terms cytokine-mediated signaling pathway and response to cytokine. These genes are depicted in a heat map, which illustrates the log_2_ fold changes of the two comparisons wt vs IFN and dox vs doxIFN (Fig 6F). This heat map includes the ISGs identified by RT-qPCR (*CCL8*, *CCL7* and *CD38*) as well as novel ATRX-regulated ISGs, including *IL8*, *CXCL1* and *CCL2*. Interestingly, only a small number of ISGs (22 genes) could be identified as differently regulated in ATRX knockdown HFFs by the RNA-seq. From these results, we concluded that ATRX specifically regulates a distinct set of ISGs upon stimulation with IFN-β. Consequently, ATRX contributes to the establishment of an antiviral state as a novel co-regulator of the IFN system.

### ATAC-seq reveals that ATRX modulates chromatin accessibility upon IFN-β stimulation

**Fig 7:**
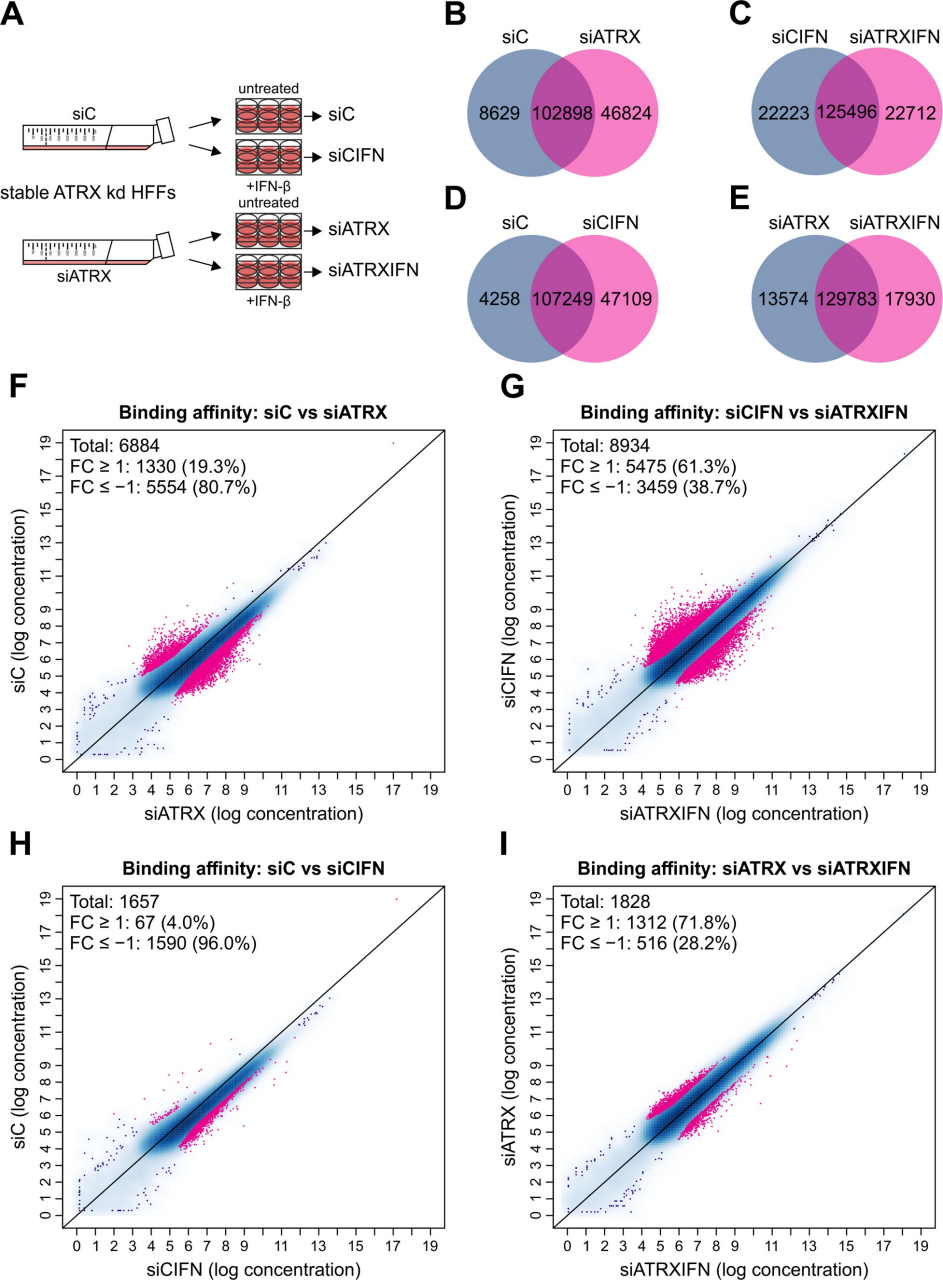
Chromatin accessibility in stable ATRX knockdown HFFs stimulated with IFN-β determined by ATAC-seq. (A) Schematic representation of the experiment. Stable ATRX knockdown HFFs (siATRX) and respective control HFFs (siC) were treated with IFN-β (2.22 × 10^3^ U/ml) for 24 h or left untreated. Cryopreserved samples were subjected to ATAC-seq. Samples were divided in four groups of duplicates (untreated control HFFs: siC; IFN-β-treated control HFFs: siCIFN; ATRX knockdown HFFs: siATRX; IFN-β-treated ATRX knockdown HFFs: siATRXIFN). (B-E) Venn diagram of common and specific peaks of the comparison (B) siC vs siATRX, (C) siCIFN vs siATRXIFN, (D) siC vs siCIFN and (E) siATRX vs siATRXIFN. (F-I) Scatter plot of common peaks considering a FDR < 0.010 for the comparison (F) siC vs siATRX, (G) siCIFN vs siATRXIFN, (H) siC vs siCIFN and (I) siATRX vs siATRXIFN. Regions with a FC ≥ 1 or a FC ≤ −1 are highlighted in pink.

Having identified that ATRX regulates expression of several ISGs, we aimed to investigate the underlying mechanism. Since ATRX is a well-known chromatin remodeler associated with both positive and negative regulation of gene expression, we were especially interested in assessing the chromatin environment in ATRX-depleted HFFs upon stimulation with IFN-β [24,25,28–30]. To address this, we conducted the assay for transposase-accessible chromatin followed by sequencing (ATAC-seq). Stable ATRX knockdown HFFs (siATRX) and respective control HFFs (siC) were stimulated with IFN-β for 24 h or left untreated, followed by ATAC-seq (Fig 7A). Samples were divided in four groups of duplicates (untreated control cells: siC; IFN-β-treated control cells: siCIFN; untreated ATRX knockdown cells: siATRX; IFN-β-treated ATRX knockdown cells: siATRXIFN) and differential binding analysis between the groups was performed. Peaks were compared between the different samples, which resulted in the identification of common and unique peaks (Fig 7B–7E). For further analyses, only common peaks between each comparison with a FDR < 0.010 and a FC ≥ 1 or a FC ≤ −1 were considered and are highlighted in pink (Fig 7F–7I). For the comparison between siC and siATRX, the analysis detected in total 6884 regions to be differentially bound between the two groups (Fig 7F). As expected, the majority of these regions (80.7%) displayed a FC ≤ −1, indicative of more accessible chromatin in the ATRX knockdown HFFs compared to the control cells. Intriguingly, this ratio shifted completely upon stimulation with IFN-β: from the 8934 common regions of the comparison siCIFN vs siATRXIFN, only 38.7% displayed a FC ≤ −1 (Fig 7G). Consequently, in the majority of the identified regions (61.3%), chromatin accessibility was reduced in ATRX knockdown HFFs treated with IFN-β compared to control cells treated with IFN-β. This matches the results of our RNA-seq, which showed that transcription of several genes is impaired in ATRX knockdown HFFs upon stimulation with IFN-β (Fig 6). For the comparison siC vs siCIFN, as expected, the vast majority (96.0%) of the 1657 common regions showed more accessible chromatin in IFN-β-treated control cells, as represented by a negative FC (Fig 7H). Remarkably, this was not the case in the ATRX knockdown cells: only 28.2% of the 1828 common regions exhibited a FC ≤ −1 (Fig 7I). Consequently, chromatin accessibility was severely reduced in IFN-β-treated ATRX knockdown HFFs compared to untreated knockdown HFFs.

**Fig 8:**
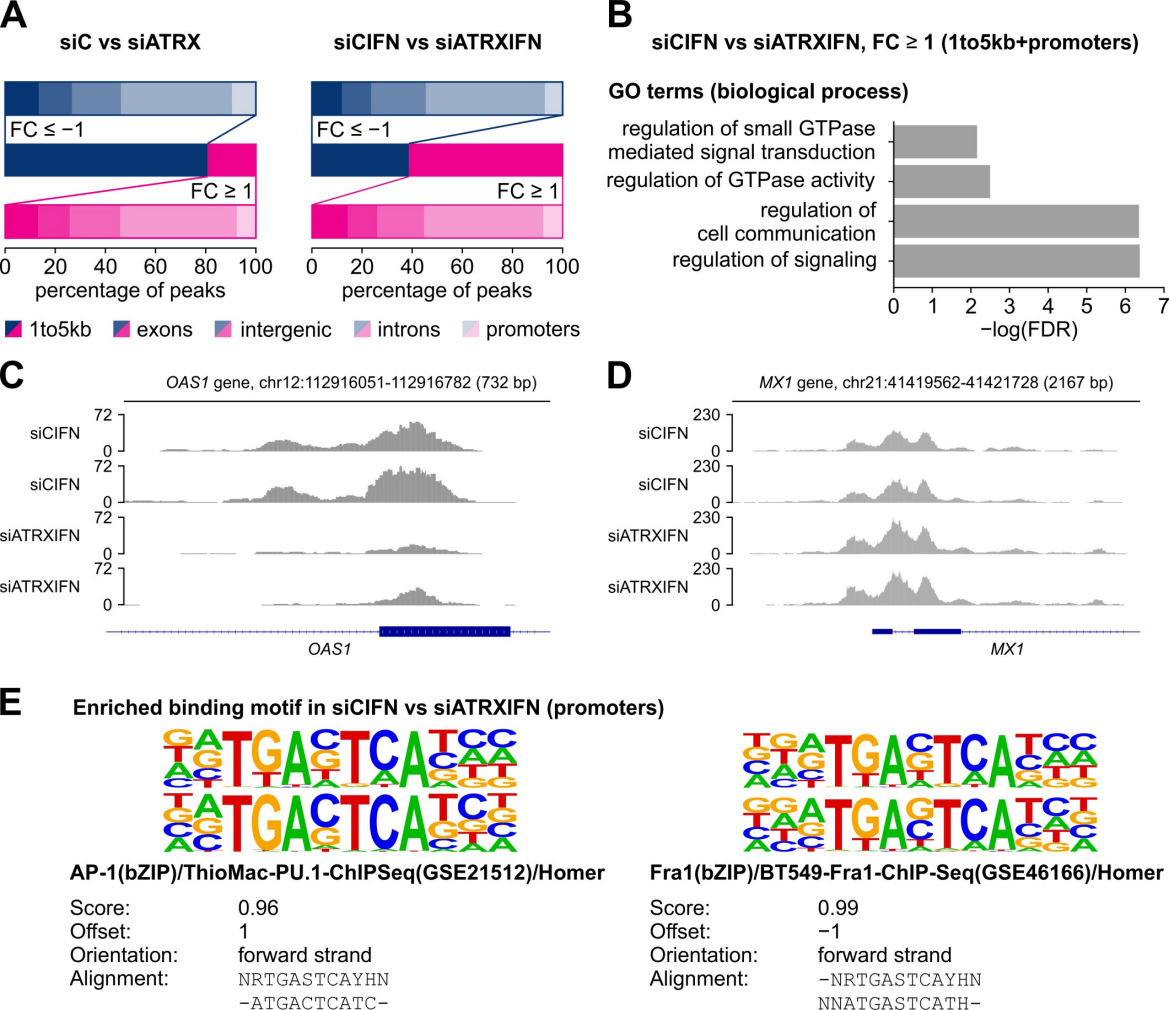
Annotation of differentially open chromatin in stable ATRX knockdown HFFs stimulated with IFN-β. (A) Gene annotations of regions with a FC ≥ 1 or a FC ≤ −1 in the comparison siC vs siATRX and siCIFN vs siATRXIFN. (B) GO-enrichment analysis of genes annotated to regions 1 to 5 kb prior to a TSS and promoter regions with a FC ≥ 1 in the comparison siCIFN vs siATRXIFN. Analysis was performed using the Gene ontology database. (C+D) Exemplary Integrative Genomics Viewer (IGV) tracks of the region annotated to (C) *OAS1* showing less accessible chromatin in siATRX cells and (D) *MX1* showing no change in chromatin accessibility. Both duplicates of the samples siCIFN and siATRXIFN are shown. (E) Homer *de novo* motif analysis of peaks annotated to promoter regions of the comparison siCIFN vs siATRXIFN. The top motif (p-value of 10^−103^) is shown (top) aligned with the matched known motifs (bottom).

In order to further investigate differentially open chromatin with regard to the location on the genome, we annotated the common regions between the comparisons siC vs siATRX and siCIFN vs siATRXIFN to the following genomic regions: 1 to 5 kb upstream of a transcription start site (TSS) (1to5kb), promoters, exons, introns and intergenic regions (Fig 8A). Interestingly, for both comparisons, changes in chromatin accessibility were evident throughout the genome (Fig 8A). Without IFN-β treatment, depletion of ATRX resulted in increased chromatin accessibility in over 80% of all regions (Fig 8A, left panel), whereas a shifted ratio was observed in all regions following IFN-β stimulation (Fig 8A, right panel). We focused further analyses on regulatory regions (regions 1 to 5 kb prior to a TSS and promoter regions), as this could represent a potential mechanism of ATRX-mediated gene regulation upon IFN signaling. Interestingly, GO enrichment analysis of genes annotated to these regions identified rather general categories and several categories related to GTPase activity (Fig 8B). In the categories regulation of cell communication and regulation of signaling, we identified the ISGs *TNFSF10*, *OAS1* and *CCL25*. As an example, ATAC-seq reads for the region annotated to *OAS1* are depicted for the comparison siCIFN vs siATRXIFN, showing reduced chromatin accessibility in siATRXIFN (Fig 8C). Interestingly, this was not the case for all ISGs, as chromatin accessibility in the region annotated to *MX1*, for example, was not altered (Fig 8D). This correlates with results of our previous RT-qPCR experiments (Fig 5B and 5E). From these results, we concluded that ATRX positively modulates chromatin accessibility specifically upon stimulation with IFN-β. Moreover, we hypothesized that these changes in chromatin accessibility in ATRX-depleted cells most likely affect the binding of transcription factors at these regions. As HOMER motif analysis of the differentially expressed genes in inducible ATRX knockdown cells suggested that ATRX regulates expression of AP-1 target genes (Fig 6C), we next aimed to investigate whether AP-1 binding could be affected in ATRX-depleted cells. Thus, we performed *de novo* motif analysis using HOMER with the peaks annotated to promoter regions from the comparison siCIFN vs siATRXIFN. Remarkably, the most significantly enriched motif indeed corresponded to the binding motif of AP-1 family members (Fig 8E). Thus, we concluded that the transcriptional changes of AP-1 target genes in ATRX-depleted cells are presumably linked to altered chromatin accessibility in their regulatory regions mediated by ATRX. Due to the well-known association of ATRX with G4 sequences and recent findings showing that G4-forming sequences are enriched at AP-1 binding sites [29,30,59], we next wondered whether G4-forming sequences at AP-1 sites might account for the observed chromatin changes in ATRX-depleted cells. Indeed, the identified AP-1 sites contained 47% more G4 sequences compared to 100 random permutations with identical peak size and number (S5A Fig). Overall, 26% of the identified AP-1 binding sites in the ATAC-seq contained at least one G4-forming sequence (S5B Fig).

**Fig 9:**
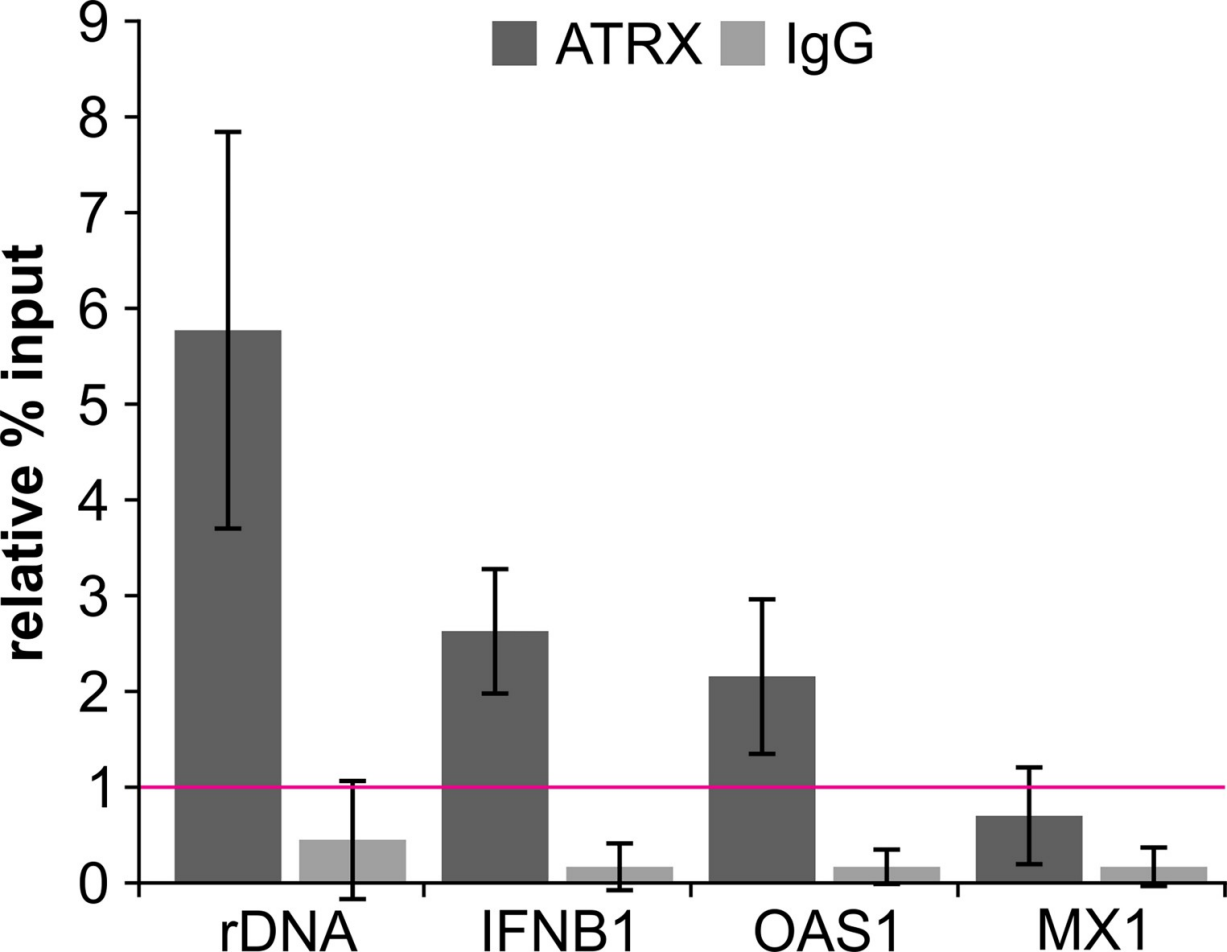
Enriched association of ATRX at target regions. HFFs were stimulated with 0.1 μg/ml poly(dA:dT) for 16 h. Cells were fixed and subjected to ChIP with an anti-ATRX antibody (D1N2E) and IgG as control. Precipitation was analyzed by qPCR using primer pairs for rDNA (positive control), IFNB1 (promoter region), OAS1 (regulatory region), MX1 (regulatory region) and GAPDH (negative control). Results are shown as the percent of input immunoprecipitated by each antibody and normalized to the negative control (GAPDH), which was set to 1 (pink line). Depicted values were calculated from three independent experiments and are shown as mean ± SD.

We next sought to test whether ATRX directly binds to some of the regulatory regions and thereby mediates chromatin accessibility as well as transcription. To analyze this, we stimulated HFFs with 0.1 μg/ml poly(dA:dT) for 16 h and performed ChIP with an ATRX antibody or IgG as control (Fig 9). Stimulation with poly(dA:dT) was used instead of IFN-β to additionally investigate a potential association of ATRX at the IFN-β promoter. Additionally, binding of ATRX to OAS1 and MX1 regulatory regions (corresponding to ATAC-seq peaks shown in Fig 8C and 8D) and rDNA as positive control was analyzed by qPCR. The background level of ATRX binding was determined using primers targeting GAPDH (Fig 9, pink line). As expected, ATRX was specifically enriched at the positive control rDNA (5.8-fold enrichment compared to GAPDH). Remarkably, we also detected an association of ATRX at the IFN-β promoter region (2.6-fold enrichment compared to GAPDH), suggesting that ATRX promotes *IFNB1* transcription via an association to the promoter region. Furthermore, we observed a 2.2-fold enrichment of ATRX at the OAS1 regulatory region, whereas no enrichment was observed for MX1. These data corroborate our ATAC-seq results and further suggest that ATRX binds to specific regions to regulate chromatin accessibility and presumably also transcription.

## Discussion

ATRX, which is a component of PML-NBs, has been demonstrated to be involved in a variety of cellular processes including intrinsic immunity against viruses and transcriptional regulation. Several studies have also revealed a role of PML-NBs in innate immune signaling [36–39]. However, most of these studies focused on the main component of PML-NBs, PML [42–45]. The potential role of ATRX in IFN signaling still remains largely unknown. Intriguingly, a recent publication implied a role of ATRX in the cytosolic cGAS-STING DNA sensing pathway in ALT positive tumor cells, by showing that loss of both STING and ATRX expression is responsible for a defective DNA sensing in ALT cells [49]. In this study, we further investigated the role of ATRX in this principal part of the innate immune system using primary cells that express an shRNA targeting ATRX. We observed that depletion of ATRX resulted in a suppressed innate immune response to HCMV infection, as detected by reduced phosphorylation of IRF3 and decreased *IFNB1* transcription (Fig 1D and 1E). However, since ATRX is known to restrict viral replication, this result did not differentiate between a direct effect of ATRX on the DNA sensing pathway or the increased infection efficiency as reason for an altered immune response. Using poly(dA:dT) as a direct activator of the DNA sensing pathway, we could confirm reduced IRF3 phosphorylation and *IFNB1* transcription in ATRX-depleted cells (Fig 2A–2C). This is consistent with observations made by Chen et al., who showed that silencing of ATRX by siRNAs resulted in diminished IRF3 phosphorylation and suppressed *IFNB1* mRNA expression in immortalized cells derived from BJ normal human fibroblasts [49]. Furthermore, we detected that depletion of ATRX affected phosphorylation and therefore activation of TBK1, the kinase upstream of IRF3 (Fig 2A). The observed effects appear to be independent of the initial DNA sensing step by cGAS, as treatment with the STING ligand 2’3’-cGAMP resulted in the same alterations as poly(dA:dT) stimulation (Fig 2D–2F). Since we observed similar effects when activating the RIG-I/MDA-5 signaling pathway via stimulation with poly(I:C) (Fig 4), we presume that ATRX activity is most likely independent of the adaptor proteins STING and MAVS, suggesting a more general role of ATRX in the production of type I IFNs. As ATRX is typically localized within the nucleus, we believe that ATRX mainly acts on nuclear events that occur during DNA and RNA sensing [21,34]. This is in line with our results showing an interaction of ATRX with IRF3 and an association with the IFN-β promoter (Figs 3G–3I and 9). Although ATRX is largely associated with heterochromatin and transcriptional repression, several studies imply a role of ATRX in activation of gene expression through association with respective gene regulatory regions [28–30]. Notably, besides transcription factors and coactivators, chromatin-remodeling proteins of the SWI/SNF family were found to be recruited to the enhanceosome [15]. Considering that ATRX belongs to the SWI/SNF family and the fact that our ChIP results showed an association of ATRX at the IFN-β promoter (Fig 9), we presume that recruitment of ATRX to the IFN-β promoter is required for an efficient transcription. However, whether ATRX is directly recruited to the promoter or the association is mediated via its interaction with IRF3, cannot be concluded from our experiments. We propose that the interaction of ATRX with IRF3 is required for an efficient formation of the IFN-β enhanceosome and furthermore retains activated IRF3 in the nucleus. Reduced phosphorylation of IRF3 in ATRX knockdown HFFs could consequently be a result of a diminished retention of IRF3 in the nucleus. Nevertheless, we also observed reduced phosphorylation of TBK1 at serine 172 in ATRX-depleted HFF upon DNA and RNA stimulation (Figs 2A, 2D and 4A), which occurs upstream of *IFNB1* transcription. Several proteins involved in DNA and RNA sensing, including cGAS, RIG-I and MDA-5, are additionally upregulated by IFNs [3,60,61]. Furthermore, activation of IRF3 is also regulated by IFNAR1 signaling, which functions as a positive feedback loop [62]. Therefore, the observed effects on IRF3 and TBK1 phosphorylation could in part be also explained by this feedback regulation due to the reduced secretion of type I IFNs in ATRX-depleted cells (Figs 2C, 2F and 4C). However, at this point, we cannot exclude other effects of ATRX involved in regulating the production of type I IFNs. Although the RNA-seq did not reveal altered transcription of genes associated with IFN signaling (S1 Table), it is still possible that ATRX depletion affects expression of kinases or phosphatases that regulate phosphorylation of TBK1 and IRF3.

We showed that depletion of ATRX results in an overall impaired antiviral state by decreasing transcription and subsequent secretion of type I IFNs, which is followed by reduced transcription of ISGs. This represents a novel regulatory function of ATRX in innate immunity. Additionally, ATRX does not only indirectly regulate ISG transcription by altering type I IFN transcription and secretion, it also has a direct effect on transcription of several ISGs induced by both IFN-β and IFN-γ (Fig 5). By generating HFFs with an inducible ATRX knockdown, we could confirm the specific regulation of several ISGs by ATRX and thereby exclude potential off-target effects arising due to the longer cultivation of cells depleted of ATRX (Fig 5E). However, it should be noted that a single siRNA target sequence was used for both the stable and inducible knockdown. A second siRNA target sequence or a different approach, such as CRISPR/Cas9-mediated knockout, would be beneficial to further validate the on-target activity. Nonetheless, RNA-seq confirmed that several ISGs are directly regulated by ATRX (Fig 6). Functional analysis of ATRX-regulated genes additionally identified genes associated with DNA replication and cell cycle regulation, which is consistent with well-described functions of ATRX [22,63–67]. Notably, the fact that RNA-seq identified a distinct set of ISGs that is regulated by ATRX further emphasizes a specific mechanism of regulation. This differs from the effect of another component of PML-NBs on ISG transcription: several studies demonstrated that PML, the main component of PML-NBs, activates the transcription of numerous ISGs induced by type I as well as type II IFNs [42–44]. Potential mechanisms include the association of PML with STAT1 and STAT2 as well as ISG promoters [44]. Since ATRX specifically targets distinct ISGs, we excluded a general effect of ATRX on STAT proteins and rather presume an association of ATRX with ISG promoter or enhancer regions. Moreover, since a knockdown of Daxx did not diminish ISG mRNA levels (S3 Fig), we ruled out that Daxx is required for ATRX-mediated regulation of ISG expression. However, we cannot entirely exclude the involvement of the ATRX-Daxx-H3.3 chromatin remodeling complex in ISG expression. ATRX and Daxx are typically required for the replication-independent deposition of H3.3 at telomers and pericentromeric heterochromatin, while the HIRA complex, composed of HIRA, Ubinuclein 1 (UBN1), Calcineurin-binding protein 1 (CABIN1) and anti-silencing function 1A (ASF1A), mediates H3.3 incorporation into actively transcribed regions [24,25,68–70]. Both histone chaperone complexes have been implicated in the regulation of intrinsic immune responses [36–39,46,47]. Moreover, it was recently shown that HIRA is recruited to PML-NBs upon activation of the IFN signaling pathway and thereby mediates innate immune responses and ISG expression [46,48]. Loading of H3.3 by HIRA on ISGs to mediate their transcription was suggested as a potential mechanism [48]. Similarly, ATRX might deposit H3.3 at specific loci to mediate expression of a distinct set of ISGs. In this case, an involvement of Daxx appears likely, since Daxx acts as the histone chaperone, while ATRX recruits Daxx to respective regions [24,25,70]. Whether other histone chaperones, such as HIRA, may compensate for the loss of Daxx, which would explain the lack of effect in Daxx knockdown cells, remains to be elucidated. As motif analysis revealed that genes regulated by ATRX upon IFN signaling could be targets of the AP-1 family of transcription factors (Fig 6C), we presume that ATRX might alter AP-1 activity to regulate gene expression. Aside from that, several studies have indicated that ATRX regulates transcription of genes by resolving G4 structures that are formed near regulatory regions of corresponding genes [29,30,64]. Therefore, another potential mechanism used by ATRX to promote ISG transcription might be the association of ATRX with G4 structures within regulatory regions of these genes. This assumption is further supported by findings of Lago et al. revealing that G4s are enriched at AP-1 binding sites and involved in transcriptional regulation at these sites [59] as well as our results showing enriched G4-forming sequences at AP-1 sites identified in the ATAC-seq (S5 Fig).

Utilizing ATAC-seq, we demonstrated significant changes of chromatin accessibility in ATRX-depleted cells upon IFN signaling (Figs 7 and 8). Without IFN-β treatment, chromatin accessibility was mostly increased upon depletion of ATRX (Fig 7F). This is consistent with recent results from Liang et al. and Teng et al., who both showed that a knockout of ATRX results in increased chromatin accessibility in human liver cancer cells and embryonic stem cells, respectively [71,72]. Thus, our data further emphasize the important function of ATRX in heterochromatin formation. Remarkably, however, upon IFN-β stimulation, ATRX knockdown HFFs were mostly associated with decreased chromatin accessibility, particular also at regions 1 to 5 kb prior to a TSS and promoter regions (Figs 7G and 8A). Interestingly, these changes in chromatin accessibility appear to be rather global and not specific to a certain pathway (Fig 8B). Consequently, we conclude that ATRX positively modulates chromatin accessibility upon IFN signaling to promote expression of ISGs as well as other genes. Moreover, we found that the binding motif of AP-1 family members was highly enriched in differentially open chromatin regions of ATRX knockdown cells treated with IFN-β (Fig 8E). This observation is consistent with previous results from Liang et al., who identified that the AP-1 motif was enriched at sites with enhanced chromatin accessibility in ATRX knockout cells [71]. With this finding, we are able to correlate altered expression of ATRX-regulated genes that were identified by RNA-seq with differential chromatin accessibility. Furthermore, considering that the AP-1 transcription factors c-Jun and ATF2 are well characterized components of the enhanceosome and thus mediate *IFNB1* transcription, it is plausible to assume that ATRX regulates chromatin accessibility at the enhanceosome, further supporting our assumption of a direct role of ATRX in *IFNB1* transcription [14–16]. Considering the results from the ChIP experiment (Fig 9), we propose that ATRX recognizes and binds to certain regulatory regions to mediate chromatin accessibility specifically in response to IFN. Based on our findings regarding G4 enrichment of AP-1 sites (S5 Fig) and the well-established association of ATRX and G4s, it is likely that G4 formation is also involved in the binding of ATRX at these regulatory regions. Whether the localization of ATRX to PML-NBs is required for these effects, as it was shown for HIRA [48], cannot be concluded from our experiments so far.

In summary, our results show that ATRX positively regulates the production of type I IFNs and specifically regulates the transcription of a set of ISGs, thereby identifying ATRX as a novel regulator of the innate immune response. Thus, we propose that the targeting of ATRX by viral regulatory proteins has evolved as an important strategy of viruses to counteract not only the intrinsic antiviral response but also innate immune signaling. Moreover, this newly uncovered role of ATRX in innate immune response is not only important in regards to virus-host interactions but may also be relevant in cancer cells that exhibit *ATRX* mutations, as it was postulated previously [49].

## Materials and methods

### Oligonucleotides and plasmids

The oligonucleotides used in this study were purchased from Biomers GmbH (Ulm, Germany), Biomol GmbH (Hamburg, Germany) and Cell Signaling Technology (Frankfurt am Main, Germany). The following oligonucleotides were used for cloning: 5’-siATRX (GATCCGAGGAAACCTTCAATTGTATTCAAGAGATACAATTGAAGGTTTCCTCTTTTTTACGCGTG), 3’-siATRX (AATTCACGCGTAAAAAAGAGGAAACCTTCAATTGTATCTCTTGAATACAATTGAAGGTTTCCTCG), 5’-miR30siATRX (TCGAGAAGGTATATTGCTGTTGACAGTGAGCGCAGAGGAAACCTTCAATTGTAATAGTGAAGCCACAGATGTATTACAATTGAAGGTTTCCTCTTTGCCTACTGCCTCGG), 3’-miR30siATRX (AATTCCGAGGCAGTAGGCAAAGAGGAAACCTTCAATTGTAATACATCTGTGGCTTCACTATTACAATTGAAGGTTTCCTCTGCGCTCACTGTCAACAGCAATATACCTTC). The following oligonucleotides were used for quantitative reverse transcription-PCR (RT-qPCR): 5’-MX1 (CAGCACCTGATGGCCTATCA), 3’-MX1 (ACGTCTGGAGCATGAAGAACTG), 5’-hISG54 (ATGTGCAACCTACTGGCCTAT), 3’-hISG54 (TGAGAGTCGGCCCATGTGATA), 5’-huGAPDH (GAAGGTGAAGGTCGGAGT), 3’-huGAPDH (GAAGATGGTGATGGGATTTC), 5’-CIITA (AACATCACTGACCTGGGTGCCTAC), 3’-CIITA (CCCACGTCGCAGATGCAGTTATTG), GAPDH Real Time PCR Primer Set (VHPS-3541, Biomol GmbH), ACTB Real Time PCR Primer Set (VHPS-110, Biomol GmbH), ATRX Real Time PCR Primer Set (VHPS-685, Biomol GmbH), CCL8 Real Time PCR Primer Set (VHPS-1625, Biomol GmbH), IRF3 Real Time PCR Primer Set (VHPS-4629, Biomol GmbH), OASL Real Time PCR Primer Set (VHPS-6424, Biomol GmbH), TNFSF10 Real Time PCR Primer Set (VHPS-9439, Biomol GmbH), IFNB1 Real Time PCR Primer Set (VHPS-4476, Biomol GmbH), CCL7 Real Time PCR Primer Set (VHPS-1624, Biomol GmbH), CD38 Real Time PCR Primer Set (VHPS-1681, Biomol GmbH). The following oligonucleotides were used for ChIP-qPCR: rDNA (#14901, Cell Signaling Technology), GAPDH (#5516, Cell Signaling Technology), 5’-IFNB1_ChIP (CAGGAGAGCAATTTGGAGGA) [45], 3’-IFNB1_ChIP (TGCTCTGGCACAACAGGTAG) [45], 5’-OAS1_ChIP (CAGCCCAGGGATTTCGGAC), 3’-OAS1_ChIP (AGCATACCTGGGTTTCGTGAG), 5’-MX1_ChIP (GACTTGCAGGAAATGCAGCC), 3’-MX1_ChIP (TCACATACACCGCTAGCACC).

For the generation of the expression plasmid encoding GFP-V5-IRF3, the V5-IRF3 coding sequence was cloned from V5-IRF3-pcDNA3 (a gift from Saumen Sarkar, Addgene plasmid #32713) [73] into pEGFP-C1 (Clontech/TaKaRa Bio, Saint-Germain-en-Laye, France) using EcoRI and XbaI.

The generation of the lentiviral expression vector pLVX-shRNA1 (Clontech, Takara, Saint-Germain-en-Laye, France) containing a control short hairpin RNA (shRNA), PML shRNA and Daxx shRNA has been described previously and the pLVX-shRNA1 plasmid containing an shRNA targeting ATRX was generated analogously [74–76]. Briefly, ATRX shRNA was generated using the oligonucleotides 5’-siATRX and 3’-siATRX and initially cloned into the retroviral vector pSIREN-RetroQ (Clontech, Takara) using BamHI and EcoRI. Subsequently, ATRX shRNA was subcloned into the lentiviral expression plasmid pLVX-shRNA1 using BamHI and XhoI.

The mutated lentiviral expression vector pInducer10-CRSmut was generated by site-directed mutagenesis of the *cis* repression signal (CRS) in the original pInducer10-mir-RUP-PheS (a gift from Stephen Elledge, Addgene plasmid #44011) as described previously [77,78]. For the generation of pInducer10-CRSmut containing an shRNA targeting ATRX, the oligonucleotides 5’-miR30siATRX and 3’-miR30siATRX were annealed and subsequently cloned into pInducer10-CRSmut using XhoI and EcoRI.

### Cell culture, infections, and titration of virus stocks

Primary human foreskin fibroblasts (HFFs) were prepared from human foreskin tissue and maintained in minimum essential medium (MEM; Gibco, Thermo Fisher Scientific, Waltham, MA, USA) supplemented with 7% fetal bovine serum (FBS; Sigma-Aldrich, Taufkirchen, Germany) and GlutaMAX (Gibco, Thermo Fisher Scientific, Waltham, MA, USA). HFFs with a stable shRNA-mediated knockdown of ATRX (siATRX) and control HFFs (siC) were maintained in MEM (Gibco, Thermo Fisher Scientific) supplemented with 10% FBS (Sigma-Aldrich, Taufkirchen, Germany), GlutaMAX (Gibco, Thermo Fisher Scientific) and 1 μg/ml puromycin (InvivoGen, Toulouse, France). HFFs with a doxycycline-inducible expression of an shRNA targeting ATRX were cultured in MEM (Gibco, Thermo Fisher Scientific) supplemented with 10% tetracycline-free FBS (Clontech, Takara, Saint-Germain-enLaye, France), GlutaMAX (Gibco, Thermo Fisher Scientific) and 1 μg/ml puromycin (InvivoGen).

Human embryonic kidney 293T (HEK293T) cells were maintained in Dulbecco’s modified Eagle medium (DMEM; Gibco, Thermo Fisher Scientific, Waltham, MA, USA) supplemented with 10% FBS (Sigma-Aldrich, Taufkirchen, Germany) and GlutaMAX (Gibco, Thermo Fisher Scientific).

Infection experiments were performed with the laboratory HCMV strain AD169. For this, HFFs were seeded into six-well plates at a density of 3 × 10^5^ cells/well one day prior to incubation with 1 ml viral supernatant. At 1.5 hours post-infection (hpi), the cells were washed, provided with 2 ml fresh medium and used for subsequent analyses. Viral titers were determined by IE1 fluorescence as described before [43].

### Reagents

Doxycycline hyclate (D9891-1G) was purchased from Sigma-Aldrich (Taufkirchen, Germany) and used at a concentration of 0.5 μg/ml. Recombinant human IFN-β (#11415–1) and IFN-γ (#285-IF-100) were purchased from R&D systems (Minneapolis, MN, USA). Poly(dA:dT)/LyoVec (#tlrl-patc), poly(I:C) high molecular weight (HMW)/LyoVec (#tlrl-piclv), poly(I:C) low molecular weight (LMW)/LyoVec (#tlrl-picwlv) and 2’3’-cGAMP (#tlrl-nacga23) were purchased from InvivoGen (Toulouse, France). Ruxolitinib (#AG-CR1-3624) was purchased from Biomol GmbH (Hamburg, Germany). Stock solutions were prepared and substances were either used directly or stored according to the manufacturers’ instructions.

### Generation of ATRX knockdown HFFs by lentiviral transduction

For the generation of stable and doxycycline-inducible ATRX knockdown HFFs, replication-deficient lentiviral particles were generated. For the stable knockdown, HEK293T cells were seeded into 10 cm dishes at a density of 5 × 10^6^ cells/dish and subsequently transfected with the lentiviral expression plasmid pLVX-shRNA1 containing either control shRNA or ATRX shRNA together with the 3rd generation packaging plasmids pLP1, pLP2 and pLP-VSVg (Invitrogen, Thermo Fisher Scientific, Waltham, MA, USA) using the transfection reagent Lipofectamine 2000 (Invitrogen, Thermo Fisher Scientific). Alternatively, in case of the inducible knockdown, HEK293T cells were transfected with the lentiviral expression plasmid pInducer10-CRSmut containing shRNA targeting ATRX together with the 2nd generation packaging plasmid pCMVdeltaR8.9 and pLP-VSVg [79]. Supernatants containing lentiviral particles were harvested 48 h after transfection, filtered (0.45 μm) to remove any remaining cells and either directly used for lentiviral transduction or stored at −80°C. HFFs of a low passage number were seeded into six-well dishes at a density of 8 × 10^4^ cells/well and incubated for 24 h with lentiviral supernatants in the presence of 7.5 μg/ml hexadimethrine bromide (Sigma-Aldrich, Taufkirchen, Germany) to increase transduction efficiency. Successfully transduced cells were selected beginning at 48 h after transduction by the addition of 1 μg/ml puromycin (InvivoGen, Toulouse, France) to the cell culture medium.

### RNA isolation and quantitative reverse transcription-PCR (RT-qPCR)

Transduced HFFs were seeded into six-well dishes at a density of 3 × 10^5^ cells/well in triplicates and subsequently either infected with AD169 or treated with different agents (IFN-β, IFN-γ, Ruxolitinib, poly(dA:dT), poly(I:C) HMW, poly(I:C) LMW or 2’3’-cGAMP). Cells were lysed using TRIzol Reagent (Invitrogen, Thermo Fisher Scientific, Waltham, MA, USA) and total RNAs from either one or two wells were isolated using the Direct-zol RNA Miniprep Kit (Zymo Research, Freiburg, Germany) following the manufacturer’s instructions. Concentration and purity of the isolated RNAs were measured spectrophotometrically. RNAs were either directly used for cDNA synthesis or stored at −80°C. Prior to cDNA synthesis, 0.3 to 1 μg RNA was treated with dsDNase (Thermo Fisher Scientific, Waltham, MA, USA) according to the manufacturer’s instructions to ensure complete removal of genomic DNA. Afterwards, first strand cDNA was synthesized using oligo(dT)_18_ and random hexamer primers provided by the Maxima First Strand cDNA Synthesis Kit for RT-qPCR (Thermo Fisher Scientific, Waltham, MA, USA). Subsequently, 2 μl of the reaction product from the first strand cDNA synthesis was mixed with Maxima SYBR Green/ROX qPCR Master Mix (2X) (Thermo Fisher Scientific, Waltham, MA, USA), nuclease-free water and primer pairs for GAPDH, CCL8, OASL, TNFSF10 and ISG54 to a total volume of 25 μl. The qPCR was performed using an Applied Biosystems 7500 Real-Time PCR System (Applied Biosystems, Thermo Fisher Scientific, Waltham, MA, USA). The thermal cycling conditions consisted of an initial denaturation step (10 min at 95°C) followed by 40 amplification cycles (15 s at 95°C, 35 s at 60°C) and a final dissociation stage to confirm specificity of the reaction. Data were analyzed using the 7500 System SDS Software v1.4. Alternatively, 2 μl of the reaction product from the first strand cDNA synthesis was mixed with the SsoAdvanced Universal SYBR Green Supermix (Bio-Rad, Feldkirchen, Germany), nuclease-free water and primer pairs for GAPDH, ACTB, ATRX, CCL8, IRF3, OASL, TNFSF10, MX1, IFNB1, CCL7, CD38, CIITA and ISG54 to a total volume of 20 μl. In this case, qPCR was performed using an AriaMx Real-Time PCR System (Agilent, Santa Clara, CA, USA) with thermal cycling conditions consisting of an initial denaturation step (30 s at 95°C) followed by 40 amplification cycles (15 s at 95°C, 30 s at 60°C) and a final dissociation stage. Data were analyzed using the AriaMx Software v1.5. Finally, relative mRNA levels were quantitated based on the 2^−ΔΔ*C*q^ method using GAPDH or ACTB as housekeeping genes [80].

### Western blotting, indirect immunofluorescence, and antibodies

For Western Blot analyses, total cell extracts were lysed with radioimmunoprecipitation assay (RIPA) buffer (1 M NaCl, 1% NP-40 substitute, 1% sodium deoxycholate, 0.2% sodium dodecyl sulfate (SDS), 50 mM Tris-HCl pH 7.4), freshly supplemented with protease inhibitors (1 mM phenylmethylsulfonyl fluoride (PMSF), 2 μg/ml aprotinin, 2 μg/ml leupeptin, 2 μg/ml pepstatin A), for 25 min on ice. Cellular debris was removed by centrifugation at 16000 × g for 10 min at 4°C and supernatants were mixed with the Laemmli-based loading buffer ROTI-Load 1 (Carl Roth, Karlsruhe, Germany), followed by boiling at 95°C for 5 min. Alternatively, cell pellets were directly resuspended in PBS and mixed with the Laemmli-based loading buffer ROTI-Load 1 (Carl Roth, Karlsruhe, Germany), followed by boiling at 95°C for 10 min. Proteins were separated by SDS polyacrylamide gel electrophoresis (SDS-PAGE) using either 8 to 12.5% Tris-Glycine or 8% Bis-Tris polyacrylamide gels and blotted onto nitrocellulose (GE Healthcare, Solingen, Germany) or PVDF membranes (Bio-Rad, Feldkirchen, Germany). Chemiluminescence detection was performed according to the manufacturer’s instructions (ECL Detection kit; Amersham Pharmacia Biotech Europe, Freiburg, Germany). Membranes were imaged with either a Fusion FX (Vilber Lourmat, Collégien, France) or a FUJIFILM Luminescent Image Analyzer LAS-1000 (FUJIFILM Europe, Düsseldorf, Germany). Images were prepared using Adobe Photoshop CS5 or Adobe Photoshop Elements 2018 and CorelDRAW X5 or CorelDRAW 2018.

For indirect immunofluorescence analyses, transduced HFFs grown on coverslips in 12-well dishes (1.2 × 10^5^ cells/well) were fixed using 4% (w/v) paraformaldehyde (PFA) for 10 min and then washed three times with phosphate-buffered saline (PBS). Subsequently, cells were permeabilized with 0.2% (v/v) Triton X-100 in PBS for 20 min at 4°C followed by five washing steps with PBS. Afterwards, cells were incubated with primary antibodies diluted in 1% (v/v) FBS in PBS for 1.5 h at 37°C, washed three times with PBS and subsequently incubated with the corresponding fluorescence-coupled secondary antibodies diluted in 1% (v/v) FBS in PBS for 45 min at 37°C. After three washing steps with PBS, the cells were mounted with VECTASHIELD Antifade Mounting Medium with DAPI (Vector Laboratories, Burlingame, CA, USA) and analyzed using a Zeiss Axio Observer.Z1 with Apotome.2. Each fluorescent dye was excited separately at a wavelength of 385 and 475 nm, and emission was detected using appropriate filters to eliminate channel overlap. Images were processed in ZEN 2.3 lite and assembled using CorelDRAW 2018.

The monoclonal antibodies anti-ATRX (#MABE1798, clone 39-f) and anti-β-actin (#A5441, clone AC-15) as well as the polyclonal antibody anti-ATRX (#HPA001906) were purchased from Sigma-Aldrich (Taufkirchen, Germany). The monoclonal antibodies anti-ATRX (#14820, clone D1N2E), anti-IgG (#3900, clone DA1E), anti-IRF3 (#11904, clone D6I4C), anti-phospho-TBK1 (Ser172) (#5483, clone D52C2) and anti-TBK1 (#3504, clone D1B4) were purchased from Cell Signaling Technology (Frankfurt am Main, Germany). The monoclonal antibody anti-phospho-IRF3 (Ser386) (#ab76493, clone EPR2346) was purchased from abcam (Berlin, Germany). The monoclonal antibodies anti-ATRX (#sc-55584, clone D-5), anti-STAT2 (#sc-1668, clone A-7) and the polyclonal antibody anti-Daxx (#sc-7152, clone M-112) were purchased from Santa Cruz Biotechnology (Dallas, TX, USA). The monoclonal antibody anti-Daxx (#TA300370, clone E94) was purchased from OriGene Technologies (Rockville, MD, USA). Alexa Fluor 488- and 555-conjugated anti-mouse/anti-rabbit secondary antibodies used for immunofluorescence were purchased from Thermo Fisher Scientific (Waltham, MA, USA) and the horseradish peroxidase-conjugated anti-mouse/anti-rabbit secondary antibodies for Western blot analyses were obtained from Dianova (Hamburg, Germany).

### Co-Immunoprecipitation (CoIP)

For CoIP experiments, HEK293T cells were seeded into 10 cm dishes at a density of 5 × 10^6^ cells/dish and were either transfected using the transfection reagent Lipofectamine 2000 (Invitrogen, Thermo Fisher Scientific) or stimulated with 0.5 μg/ml poly(I:C) HMW. After 24 h, cells were lysed for 20 min at 4°C in 500 to 900 μL of CoIP buffer (50 mM Tris-HCl pH 8.0, 150 mM NaCl, 5 mM EDTA, 0.5% NP-40) freshly supplemented with protease inhibitors (1 mM PMSF, 2 μg/mL aprotinin, 2 μg/mL leupeptin, and 2 μg/mL pepstatin). Cellular debris was removed by centrifugation at 16000 × g for 10 min at 4°C and input samples were taken for each precipitation. The remaining supernatant was incubated with anti-ATRX (#14820, clone D1N2E) antibody coupled to protein A-Sepharose beads (Sigma-Aldrich, Taufkirchen, Germany) or protein G-Dynabeads (Thermo Fisher Scientific, Waltham, MA, USA) for 1.5 h at 4°C. Additionally, beads coupled to an IgG isotype control served as negative control. Subsequently, beads were washed five times with CoIP buffer including protease inhibitors. Finally, beads and input control were mixed with a Laemmli-based loading buffer, followed by boiling at 95°C for 10 min. Samples were analyzed by Western blotting.

### Type I interferon (IFN) secretion assay

Human type I IFNs in the supernatant of transduced HFFs were measured using HEK-Blue IFN-α/β cells purchased from InvivoGen (Toulouse, France). These cells possess a reporter gene that expresses a secreted embryonic alkaline phosphatase (SEAP) in the presence of type I IFNs. The assay was conducted according to the manufacturer’s protocol. Briefly, HEK-Blue IFN-α/β cells were seeded into 96-well plates at a density of 5 × 10^4^ cells/well and supernatants from transduced HFFs treated with poly(dA:dT), poly(I:C) HMW or 2’3’-cGAMP were added. After an incubation overnight at 37°C (5% CO_2_), supernatants from induced HEK-Blue IFN-α/β cells were mixed with QUANTI-Blue solution and incubated for 1 h at 37°C. Finally, SEAP levels were determined using a spectrophotometer at 620 nm.

### Chromatin immunoprecipitation (ChIP)

ChIP was performed utilizing the SimpleChIP Enzymatic Chromatin IP Kit (Cell Signaling Technology, Frankfurt am Main, Germany) following the manufacturer’s instructions. Briefly, HFFs (4 × 10^6^ cells for each immunoprecipitation) were treated with 0.1 μg/ml poly(dA:dT) for 16 h. Cells were fixed with formaldehyde (1% final concentration) for 10 min at room temperature. Fragmented chromatin was prepared using Micrococcal Nuclease followed by sonication. Subsequently, chromatin immunoprecipitation was performed with anti-ATRX (#14820, clone D1N2E) and anti-IgG (#2729). Cross-linking was reversed by a proteinase K digest overnight. Finally, DNA was purified and subjected to qPCR using the SsoAdvanced Universal SYBR Green Supermix (Bio-Rad, Feldkirchen, Germany) and primer pairs for the *IFNB1* promoter region (IFNB1), *OAS1* regulatory region (OAS1), *MX1* regulatory region (MX1), rDNA as a positive control and GAPDH as a negative control. The qPCR was performed using an AriaMx Real-Time PCR System (Agilent, Santa Clara, CA, USA) with thermal cycling conditions consisting of an initial denaturation step (2 min at 95°C) followed by 40 amplification cycles (15 s at 95°C, 30 s at 60°C) and a final dissociation stage. Data were analyzed using the AriaMx Software v1.5 and were calculated as percent of the total input chromatin for each immunoprecipitation.

### Whole transcriptome sequencing (RNA-seq)

For whole transcriptome sequencing, HFFs with a doxycycline-inducible expression of an shRNA targeting ATRX were treated with doxycycline for 14 d or left untreated as control. Subsequently, cells were seeded into six-well plates at a density of 3 × 10^5^ cells/well in triplicates one day prior to IFN-β stimulation for 24 h. Total RNAs from two wells per sample were isolated as described before. RNA sequencing was performed at Macrogen (Amsterdam, Netherlands) using the NovaSeq 6000 sequencing platform to generate 2 × 100 bp paired-end libraries. For primary and secondary analysis, reads were aligned to hg19 reference using bowtie2 (version 2.3.3.1). samtools (version 1.9) was used to convert sam-files into sorted bam-files. Using htseq-count (version 0.9.1) and required hg19 gtf file, readcounting was performed. Raw counts for all samples were grouped by condition and processed by DESeq2 for differential expression analysis. Differentially expressed genes were submitted to the STRING database (version 11.0) to identify an association network and perform functional enrichment analysis [58]. The *de novo* motif analysis of differentially expressed genes was performed using Hypergeometric Optimization of Motif Enrichment (HOMER) [81,82]. We used default parameters and investigated the promoter regions within −500 to 100 bp relative to the transcription start site (TSS).

### Assay for transposase-accessible chromatin followed by sequencing (ATAC-seq)

For ATAC-seq, stable ATRX knockdown HFFs (siATRX) and respective control HFFs (siC) were seeded into six-well plates at a density of 3 × 10^5^ cells/well in duplicates one day prior to IFN-β stimulation for 24 h. Subsequently, cells from three wells per sample were harvested and cryopreserved with freezing medium containing 60% RPMI-1640 (v/v) and 40% DMSO (v/v) supplemented with 12% d(+)-glucose (w/v). ATAC-seq was carried out by Diagenode SA (Seraing (Ougrée), Belgium) utilizing the ATAC-seq service (Assay for Transposase-Accessible Chromatin) (Diagenode Cat# G02060000). Briefly, 5 × 10^4^ cells were centrifuged at 500 × g followed by isolation of the nuclei using 0.4% of Igepal CA-630 for 3 min. Subsequently, the transposition reaction was carried out for 30 min at 37°C using the prokaryotic Tn5 transposase system (Nextera DNA library kit, FC-121–1030, Illumina Inc., San Diego, CA, USA) followed by purification on Diapure columns (C03040001). Library preparation was performed using the NEBNext High-Fidelity PCR MasterMix (M0541, NEB, Ipswich, MA, USA) and Illumina indexing primers, and qPCR was performed on LightCycler 96 System (Roche, Basel, Switzerland). Size selection and purification of the libraries was carried out using Agencourt AMPure XP (Beckman Coulter, Brea, CA, USA). Afterwards, libraries were quantified using Qubit dsDNA HS Assay Kit (Q32854, Thermo Fisher Scientific, Waltham, MA, USA) and their fragment size was analyzed by High Sensitivity DNA Analysis Kits on a High Sensitivity NGS Fragment Analysis Kit (DNF-474) on a Fragment Analyzer (Advanced Analytical Technologies, Inc., Agilent Technologies, Santa Clara, CA, USA). Finally, pooled libraries were sequenced on an Illumina NovaSeq 6000 with paired-end reads of 50 bp length. Standard and comparative analysis was carried out by Diagenode SA (Seraing (Ougrée), Belgium) as follows. Sequencing quality was assessed using FastQC and reads were trimmed using either cutadapt or Trim_Galore! [83]. Trimmed reads were aligned to the reference genome hg38 obtained from the UCSC genome browser [84]. After exclusion of regions blacklisted by the ENCODE project as well as the removal of PCR duplicates and multimapping reads using samtools, peaks were called using Epic2 with optimized parameters for ATAC-Seq [85–87]. Differentially open chromatin was determined using the R/Bioconductor package DiffBind [88,89]. Additionally, gene ontology (GO) enrichment analysis was performed with differentially open chromatin regions using the GO Ontology database (DOI: 10.5281/zenodo.5080993, released 2021-07-02) [90,91]. For this, the PANTHER Overrepresentation Test (Released 2021-02-24) was selected as analysis tool and Fisher’s exact test with FDR correction was performed. For visualization of ATAC-seq peaks, the integrative genomics viewer (IGV) was used [92]. The *de novo* motif analysis of differentially open chromatin regions was performed using Hypergeometric Optimization of Motif Enrichment (HOMER) with default parameters [81].

Prediction of G4 sequences and subsequent enrichment analysis were performed as described before [72]. First, a FASTA file was generated from the list of peaks identified by HOMER to contain an AP-1 motif using the BEDTools tool getfasta (v2.30.0) and hg38 as a reference. Next, a Python script (fastaRegexFinder.py (v0.2.0), https://github.com/dariober/bioinformatics-cafe/tree/master/fastaRegexFinder) was utilized to search for putative G4 sequences on the forward and reverse strand of the obtained FASTA file. The regex was defined as: “([gG]{3,}\w{1,12}){3,}[gG]{3,}”. The peaks were then divided into the following two groups: Peaks containing at least one G4 and peaks containing no G4. In order to identify whether the number of G4-containing peaks is enriched in the peaks that were found to contain AP-1 motifs (comparison siCIFN vs siATRXIFN), a simulation was carried out. For this, the peaks were randomly permuted 100 times among the hg38 genome using the BEDTools tool shuffle (v2.30.0), and the number of G4 sequences was analyzed for each run as described above. In this process, the number of peaks and length of the peaks were maintained throughout the runs.

## Supporting information

S1 Fig Reduced IRF3 and TBK1 phosphorylation in ATRX knockdown HFFs. Quantification of Western blots presented in Fig 2A, Fig 2B and Fig 4A. Stable ATRX knockdown HFFs (siATRX) and respective control HFFs (siC) were treated with (A+B) 0.1 μg/ml poly(dA:dT), (C+D) 50 μg/ml 2’3’-cGAMP or (E+F) 0.1 or 0.5 μg/ml poly(I:C) HMW for 24 h. Signal intensities were quantified relative to siC cells. Data were obtained from (A) five, (B) four or (C-F) three independent experiments and are shown as mean ± SD. (E+F) Treatment with 0.1 μg/ml and 0.5 μg/ml poly(I:C) HMW was considered as replicates. Statistical analysis was performed using a student’s *t*-test (one sample, two-tailed); *p<0.05, **p<0.01.
(TIF)

S2 Fig No effect on IRF3 protein and mRNA levels in ATRX knockdown HFFs. (A+B) Stable ATRX knockdown HFFs (siATRX) and respective control HFFs (siC) were treated with 0.1 μg/ml poly(dA:dT) for 24 h. (A) Cells were harvested for Western blot analyses to determine IRF3 protein levels. Signal intensities were quantified relative to siC cells. Data were obtained from four independent experiments and are shown as mean ± SD. Statistical analysis was performed using a student’s *t*-test (one sample, two-tailed); n.s. = not significant. (B) Total RNAs were prepared and RT-qPCR was performed to determine *IRF3* mRNA levels. Depicted values were calculated from triplicates relative to untreated siC cells using *GAPDH* as a housekeeping gene and are shown as mean ± SD. One out of three independent experiments is shown. Statistical analysis was performed with respective ΔCq-values using a student’s *t*-test (unpaired, two-tailed); n.s. = not significant.
(TIF)

S3 Fig Knockdown of ATRX, Daxx and PML affects ISG expression to different extents. Stable ATRX (siATRX), Daxx (siDaxx) and PML (siPML) knockdown HFFs and respective control HFFs (siC) were stimulated with IFN-β (2.28 × 10^3^ U/ml) for 24 h. Total RNAs were prepared and RT-qPCR was performed to determine transcription of the ISGs *ISG54*, *OASL*, *CCL8* and *MX1*. Depicted values were calculated from triplicates relative to untreated siC cells using *GAPDH* as a housekeeping gene and are shown as mean ± SD. One out of two independent experiments is shown. Statistical analysis was performed with respective ΔCq-values using a student’s *t*-test (unpaired, two-tailed); n.s.: not significant, **p<0.01, ***p<0.001.
(TIF)

S4 Fig Protein interaction network of ATRX-regulated genes using the STRING database. From the top 100 hits with reduced log_2_ fold changes in ATRX-depleted cells treated with IFN-β (dox vs doxIFN) compared to control cells treated with IFN-β (wt vs IFN), 85 corresponding proteins were analyzed using the STRING database (https://string-db.org/, accessed 22 Jan 2021). The confidence cutoff for displaying interaction links has been set to medium (0.400). The edges indicate both functional and physical protein associations. The line color indicates the type of interaction evidence.
(TIF)

S5 Fig Occurrence of G4s is enriched in AP-1-containing peaks. ATAC-seq peaks from the comparison siCIFN vs siATRXIFN identified by HOMER analysis to contain AP-1 sites (see Fig 8E) were further analyzed on potential G4 sequences. (A) The histogram shows the number of G4 sequences found in 100 random permutations among the hg38 genome in comparison to the number of G4 sequences identified in the ATAC-seq peaks (63 G4s, vertical solid line). The dashed line represents the average of identified G4 sequences in the simulation (43 G4s) with the standard deviation displayed as the grey background. (B) The pie chart represents the number of identified AP-1 sites (ATAC-seq peaks) that contain a G4 motif.
(TIF)

S1 Table Log2 fold changes in ATRX knockdown HFFs compared to control HFFs. The top 50 differentially expressed genes upon depletion of ATRX (wt vs dox) (adjusted p-value < 0.01) are listed. Non-coding RNAs are depicted in grey.
(XLSX)

S2 Table Log2 fold changes in control and ATRX knockdown HFFs upon IFN-β stimulation. Differentially expressed genes upon IFN-β stimulation in control cells (wt vs IFN) and ATRX knockdown cells (dox vs doxIFN) (adjusted p-value < 0.01) as illustrated in Fig 6D. The top 100 hits with an attenuated induction in ATRX knockdown cells compared to control cells are listed. Non-coding RNAs are depicted in grey.
(XLSX)
