# Supplementary figures and images for: The chromatin remodeling protein ATRX positively regulates IRF3-dependent type I interferon production and interferon-induced gene expression
Source: PLoS Pathog. 2022 Aug 8;18(8):e1010748. doi: 10.1371/journal.ppat.1010748 (PMC9387936; doi:10.1371/journal.ppat.1010748)

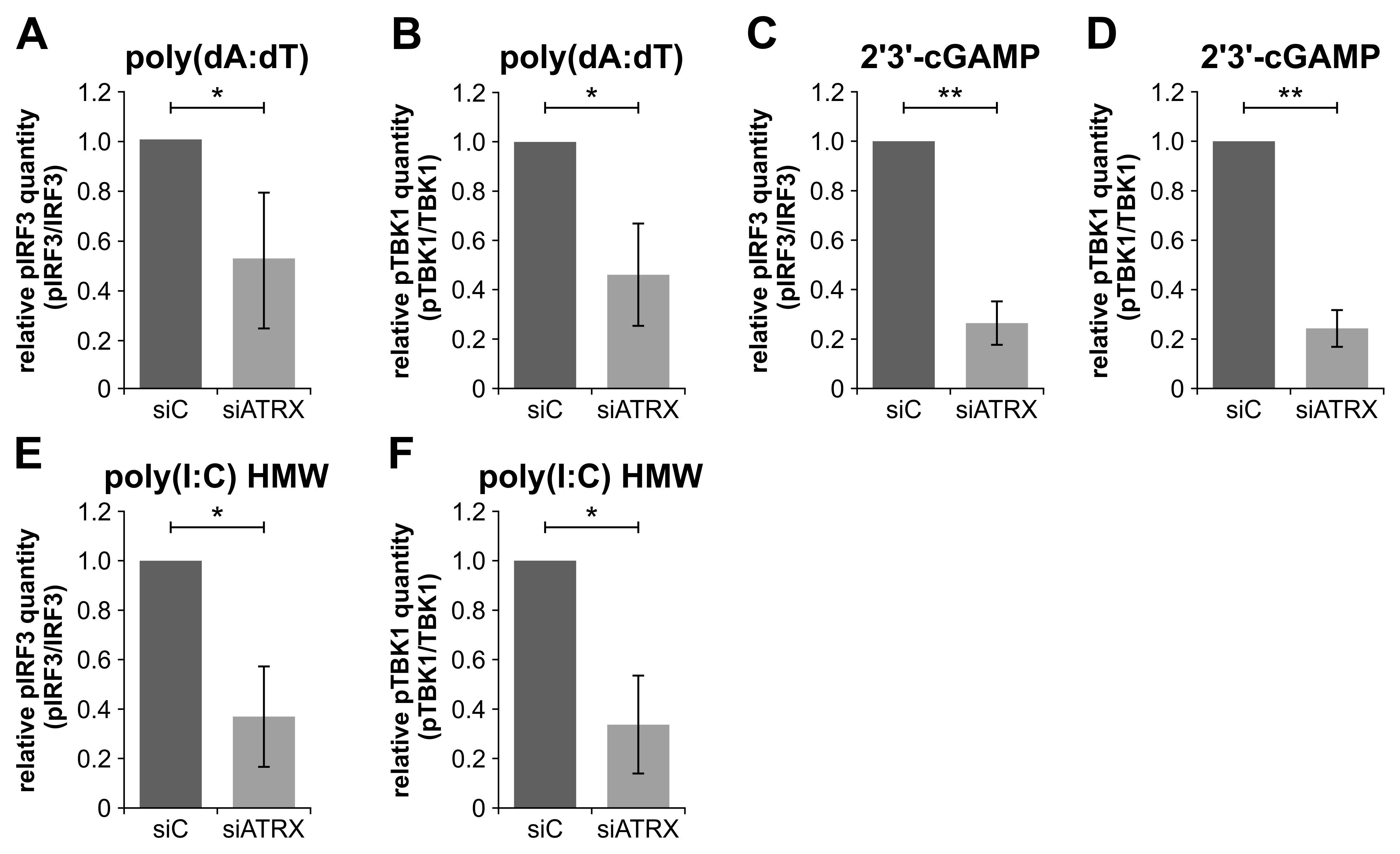

Supplement: S1 Fig — Quantification of Western blots presented in Fig 2A, Fig 2B and Fig 4A. Stable ATRX knockdown HFFs (siATRX) and respective control HFFs (siC) were treated with (A+B) 0.1 μg/ml poly(dA:dT), (C+D) 50 μg/ml 2’3’-cGAMP or (E+F) 0.1 or 0.5 μg/ml poly(I:C) HMW for 24 h. Signal intensities were quantified relative to siC cells. Data were obtained from (A) five, (B) four or (C-F) three independent experiments and are shown as mean ± SD. (E+F) Treatment with 0.1 μg/ml and 0.5 μg/ml poly(I:C) HMW was considered as replicates. Statistical analysis was performed using a student’s t-test (one sample, two-tailed); *p<0.05, **p<0.01. (TIF) [file ppat.1010748.s001.tif]

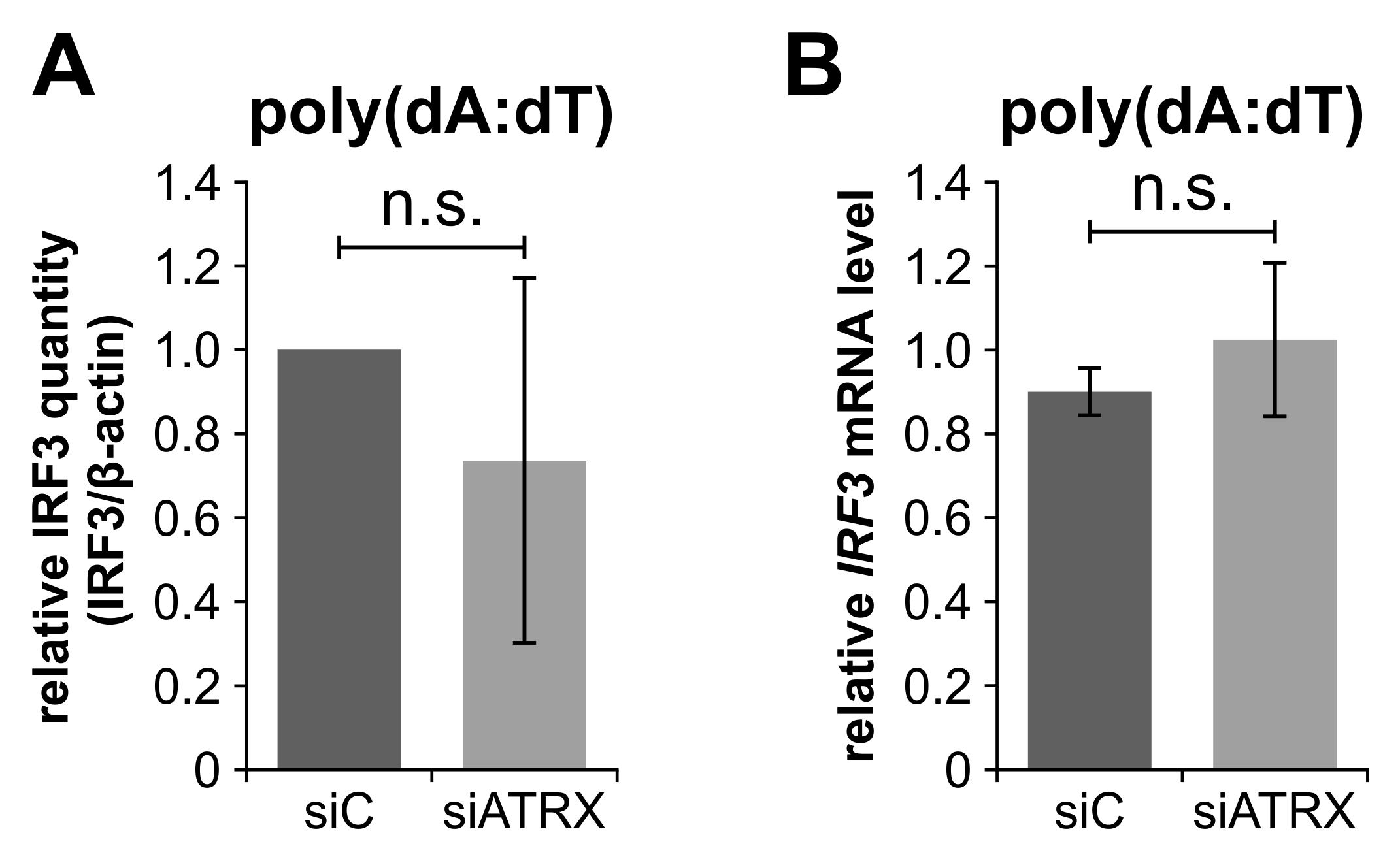

Supplement: S2 Fig — (A+B) Stable ATRX knockdown HFFs (siATRX) and respective control HFFs (siC) were treated with 0.1 μg/ml poly(dA:dT) for 24 h. (A) Cells were harvested for Western blot analyses to determine IRF3 protein levels. Signal intensities were quantified relative to siC cells. Data were obtained from four independent experiments and are shown as mean ± SD. Statistical analysis was performed using a student’s t-test (one sample, two-tailed); n.s. = not significant. (B) Total RNAs were prepared and RT-qPCR was performed to determine IRF3 mRNA levels. Depicted values were calculated from triplicates relative to untreated siC cells using GAPDH as a housekeeping gene and are shown as mean ± SD. One out of three independent experiments is shown. Statistical analysis was performed with respective ΔCq-values using a student’s t-test (unpaired, two-tailed); n.s. = not significant. (TIF) [file ppat.1010748.s002.tif]

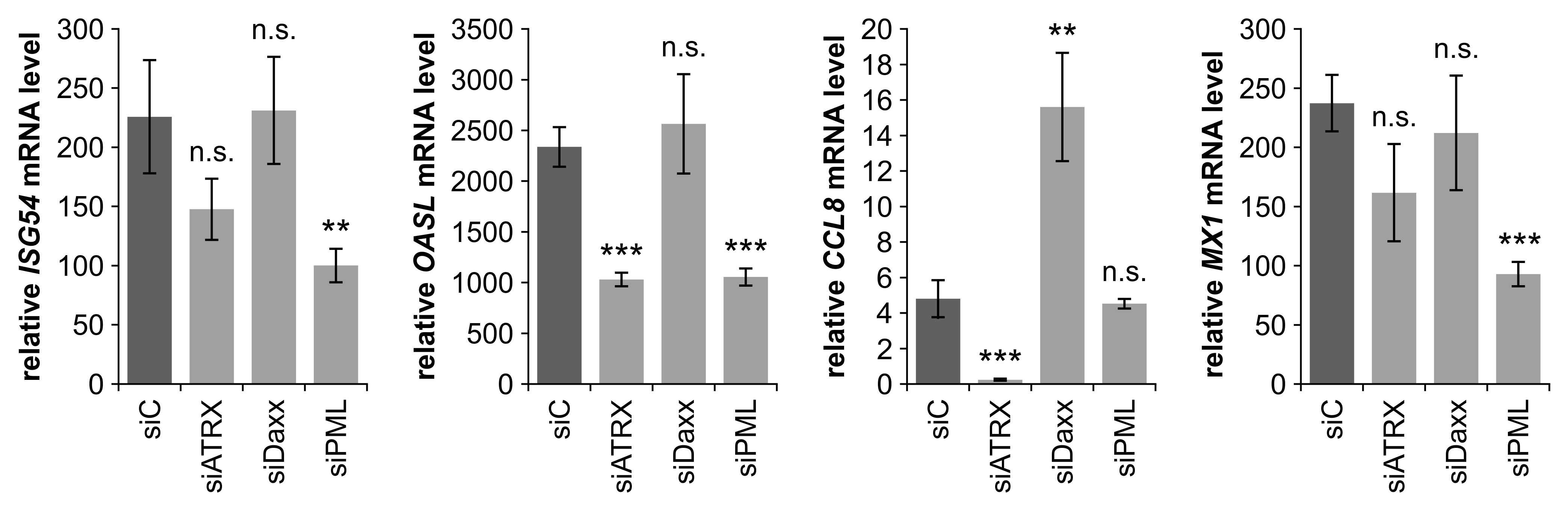

Supplement: S3 Fig — Stable ATRX (siATRX), Daxx (siDaxx) and PML (siPML) knockdown HFFs and respective control HFFs (siC) were stimulated with IFN-β (2.28 × 10^3^ U/ml) for 24 h. Total RNAs were prepared and RT-qPCR was performed to determine transcription of the ISGs ISG54, OASL, CCL8 and MX1. Depicted values were calculated from triplicates relative to untreated siC cells using GAPDH as a housekeeping gene and are shown as mean ± SD. One out of two independent experiments is shown. Statistical analysis was performed with respective ΔCq-values using a student’s t-test (unpaired, two-tailed); n.s.: not significant, **p<0.01, ***p<0.001. (TIF) [file ppat.1010748.s003.tif]

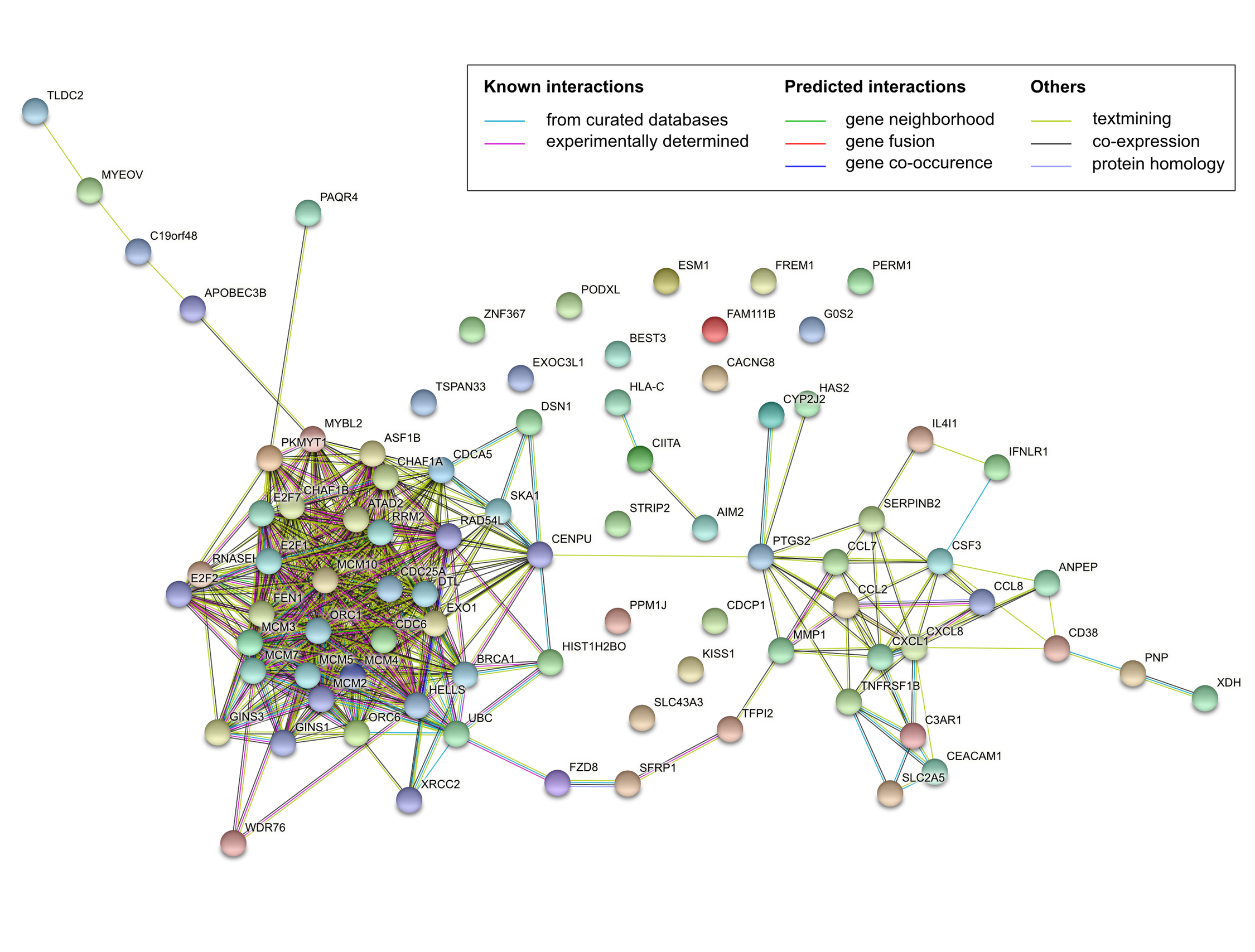

Supplement: S4 Fig — From the top 100 hits with reduced log2 fold changes in ATRX-depleted cells treated with IFN-β (dox vs doxIFN) compared to control cells treated with IFN-β (wt vs IFN), 85 corresponding proteins were analyzed using the STRING database (https://string-db.org/, accessed 22 Jan 2021). The confidence cutoff for displaying interaction links has been set to medium (0.400). The edges indicate both functional and physical protein associations. The line color indicates the type of interaction evidence. (TIF) [file ppat.1010748.s004.tif]

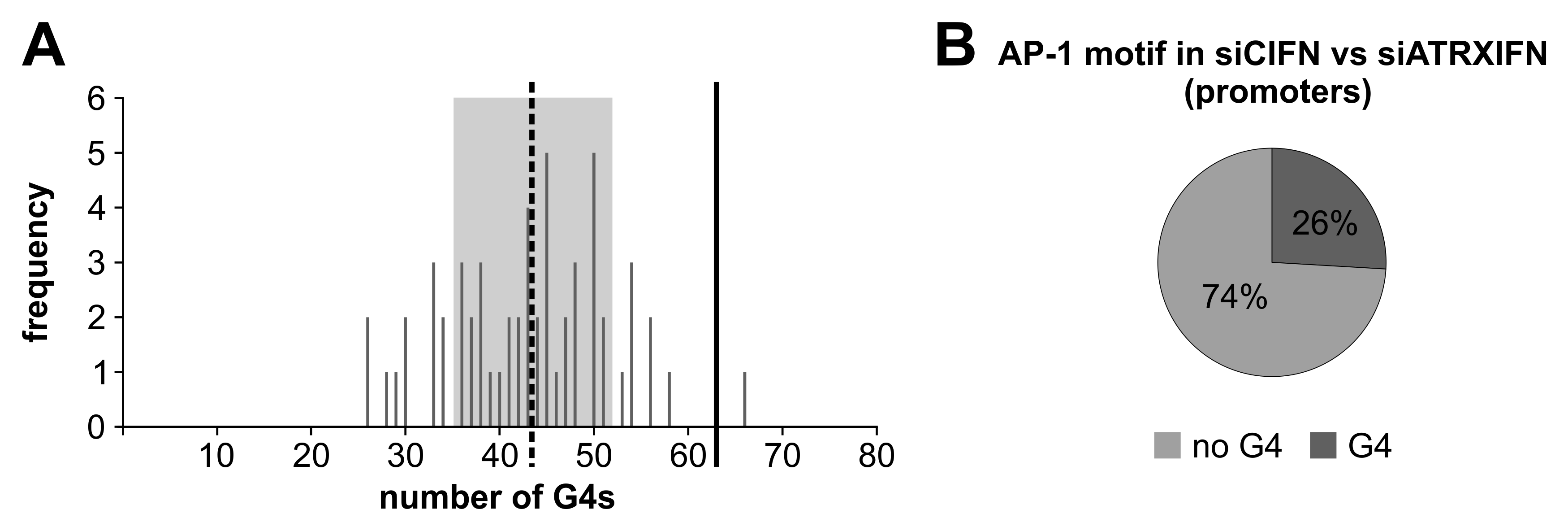

Supplement: S5 Fig — ATAC-seq peaks from the comparison siCIFN vs siATRXIFN identified by HOMER analysis to contain AP-1 sites (see Fig 8E) were further analyzed on potential G4 sequences. (A) The histogram shows the number of G4 sequences found in 100 random permutations among the hg38 genome in comparison to the number of G4 sequences identified in the ATAC-seq peaks (63 G4s, vertical solid line). The dashed line represents the average of identified G4 sequences in the simulation (43 G4s) with the standard deviation displayed as the grey background. (B) The pie chart represents the number of identified AP-1 sites (ATAC-seq peaks) that contain a G4 motif. (TIF) [file ppat.1010748.s005.tif]
